# Characterisation of Endogenous Peptides Present in Virgin Olive Oil

**DOI:** 10.3390/ijms23031712

**Published:** 2022-02-02

**Authors:** Eduardo Lopez-Huertas, Juan M. Alcaide-Hidalgo

**Affiliations:** 1 Group of Antioxidants and Free Radicals in Biotechnology, Food and Agriculture, Estación Experimental Zaidín, Consejo Superior de Investigaciones Científicas (CSIC), Profesor Albareda 1, 18008 Granada, Spain; 2 Eurecat, Technology Centre of Catalonia, Nutrition and Health Unit, 43204 Reus, Spain; juanmaria.alcaide@eurecat.org

**Keywords:** virgin olive oil, food peptides, bioactive peptides, peptidomics, angiotensin-converting enzyme, antioxidant

## Abstract

The low molecular weight peptide composition of virgin olive oil (VOO) is mostly unknown. We aimed to investigate the composition of the endogenous peptides present in VOO, the protein sources from which those peptides originate and their biological activities. A water-soluble extract containing peptides was obtained from VOO. The peptides were separated by size-exclusion using fast protein liquid chromatography, and the low molecular weight fraction (1600–700 kDa) was analysed by nanoscale liquid chromatography Orbitrap coupled with tandem mass spectrometry and de novo sequencing. Nineteen new peptides were identified by Peaks database algorithm, using the available *Olea europaea* (cv. Farga) genome database. Eight new peptides were also identified by Peaks de novo sequencing. The protein sources of the peptides detected in the database by Peaks DB were identified by BLAST-P search. Seed storage proteins were among the most frequent sources of VOO peptides. BIOPEP software was used to predict the biological activities of peptides and to simulate (in silico) the proteolytic activity of digestive enzymes on the detected peptide sequences. A selection of synthetic peptides was obtained for investigation of their bioactivities. Peptides VCGEAFGKA, NALLCSNS, CPANGFY, CCYSVY and DCHYFL possessed strong ACE-inhibitory and antioxidant activities in vitro. Antioxidant peptides could play a role in VOO quality.

## 1. Introduction

Virgin olive oil (VOO) is the principal oil consumed by Mediterranean countries [1]. VOO mainly contains fatty acids in the form of triglycerides and also a fraction (1–3%) of minor components, or micronutrients. This fraction primarily consists of polyphenols, tocopherols, sterols, terpenic acids, chlorophyll and carotenoids [2,3], which are transferred from olives to the VOO during its extraction. VOO can be consumed as such after it is produced and does not require refining procedures to eliminate undesirable compounds, therefore these micronutrients remain present in the oil and make a significant contribution to the oil’s quality and healthy properties. For example, VOO antioxidant compounds, such as certain polyphenols (hydroxytyrosol, tyrosol, oleuropein and its derivatives), protect VOO from oxidation and possess beneficial effects on cardiovascular health by protecting blood lipids from oxidative free radicals [4]. In recent years, proteins and peptides have also been described as part of a group of olive oil micronutrients [5]. However, the effects of the composition and the functions that peptides and proteins may have on the quality of VOO and their possible health benefits have been poorly studied. 

Food proteins from plant and animal sources have been used to obtain a wide range of peptides with biological activity. Previous research studies and clinical trials in humans have shown a good number of health benefits produced by food-derived peptides, including antihypertensive [6], anticancer [7,8,9], hypocholesterolemic [10,11], hypotryglyceridemic [12], antioxidant [13,14,15], anti-diabetic [16,17], anti-inflammatory [18,19], immunostimulating [20,21], antithrombotic [22,23], antiobesity [24,25], neuromodulatory and opioid [26,27].

High blood pressure is a primary and controllable risk factor of cardiovascular disease that affects approximately 40% of adults [28]. The angiotensin-converting enzyme (ACE) of the renin–angiotensin system plays a key role in regulating blood pressure. Inhibition of ACE is a widely used strategy for the treatment of hypertension [29]. A considerable number of low molecular weight peptides from foods have shown ACE-inhibitory activity. They are mostly produced via enzyme hydrolysis of proteins from animal or vegetable origins [30], although they can also occur naturally [31,32]. A well-known example of ACE-inhibitory peptides with antihypertensive activity are VPP and IPP, derived from milk casein. A recent meta-analysis including 30 randomised controlled clinical trials in which VPP and IPP were administered to human subjects concluded that the supplementation of pre- or hypertensive humans with VPP and IPP for longer than four weeks resulted in a significant reduction in systolic and diastolic blood pressure [33]. Indeed, food-derived peptides may have enormous potential for health and therapy by influencing the metabolic reactions directly involved in risk factors for chronic diseases [34].

In previous work carried out in our laboratory, we showed that unfiltered VOO contains peptides with ACE-inhibitory activity and antihypertensive activity [35]. However, the sequences of the detected low molecular weight peptides were only established preliminarily with the tools available [36]. In this study, we have used the recently available genome database and de novo sequencing assisted database (DB) search to investigate the composition of the endogenous peptides present in VOO, the protein sources from which those peptides originate and their biological activities.

## 2. Results and Discussion

### 2.1. Endogenous Peptides Identified by Peaks DB in VOO

In this study, the composition of low molecular weight peptides, as well as their ACE-inhibitory and antioxidant activities, were investigated in a water-soluble extract of unfiltered VOO. Our previous results showed that unfiltered VOO may contain between 0.3 and 1.2 mg of proteins per 100 g of oil [36]. ACE-inhibitory and antihypertensive peptides typically have molecular weights below 3000 Da [37], therefore, we focused the analysis on the low molecular weight fractions of VOO peptides. A widely accepted method for peptide and protein identification is to match MS/MS data to sequenced genomes or by de novo sequencing with software packages, such as PEAKS [38]. Using the available *Olea europaea* genome database, Peaks DB software identified 19 endogenous peptides in VOO with −10 lgP score > 30 that were not reported before. Table 1 shows a list of the identified peptide sequences and a selection of peptide parameters, including suggested protein sources (BLASTP) and accession numbers for the proteins containing the peptides. Peptide no. 19 (CPANGFY) with a −10 lgP score of 29.54 was also included in the list due its potential biological activity (peptides reported by Peaks DB with a −10 lgP score > 25 are shown in Supplementary Materials Table S1).

The identified peptides possessed between 7 and 23 amino acids and molecular masses ranging from 759 to 2673 Da. Peptide nos. 2, 3, 6 and 10 are related as follows: The amino acid sequences of peptides nos. 2, 6 and 10 are contained within the sequence of peptide no. 3, and the sequences of peptide nos. 2, 6 and 10 overlap. As expected, a high proportion of hydrophobic amino acids were detected in the sequences. Interestingly, the presence of hydrophobic or basic charged amino acids, such as arginine or lysine, was also detected within the last three amino acids of the C-terminal position for peptides no. 1–10, 12–14, 17 and 19. ACE-inhibitory peptides usually contain hydrophobic and/or basic charged amino acids at the C-terminal position [39]. In addition, most antioxidant peptides described in the literature present a high content of histidine and hydrophobic amino acids [40]. Peptide nos. 7, 14, 17, 18 and 19 possessed cysteine residues, which are often involved in electron transfer reactions with antioxidant potential [41].

### 2.2. Olive Proteins Detected as Sources of VOO Peptides

A total of 20 proteins from 13 protein groups were reported as sources of the 19 peptide sequences identified by Peaks DB (Table 2). The protein coverage and fragmentation spectra of the 19 supporting peptides are shown in Supplementary Materials Table S2. The *Olea europaea* database used in these analyses was unannotated, so the accession numbers for the proteins containing the peptides were obtained by Peaks DB, but potential protein identification was not provided. BLAST searches against the non-redundant protein sequences’ database at NCBI [42] were carried out in order to identify the protein sources of the peptides detected in VOO. 

The sequence of peptide no. 1 was identified as part of the 11S globulin subunit beta, a seed storage protein that has been described in the protein bodies of mature olive seeds [43]. Peptide nos. 2, 3, 5, 6, 10 and 12 were identified as part of legumin A-like protein, which is another kind of major globulin-type seed storage protein. In agreement with this, a recent study investigated the protein composition of VOO via (1-dimension) polyacrylamide gel electrophoresis of extracted proteins and further LC-MS analysis of the sliced protein bands obtained in the gels. The authors identified seed storage proteins of globulin-type as the most abundant proteins in VOO, including several 11S globulins, also known as legumins [44]. The presence of several peptide sequences derived from legumin-A and 11S globulin indicate that legumins are an important source of peptides in VOO. Legumin-A proteins, found in the seeds of many leguminous and non-leguminous plants, are also a source of sulphur-containing amino acids [45]. The sequences of peptide nos. 4 and 8 are associated with apyrase-like proteins, which catalyse ATP hydrolysis in cells. Peptide no. 9 is derived from a zinc transporter 5-like protein, which presents high homology with other plant zinc transporters. Peptide no. 11′s sequence is derived from L-type lectin receptor kinase S.7, a plant kinase described to be involved in defence response and pollen formation [46]. Peptide no. 13 originates from protein homolog R1A-10, an ATP-binding protein that has been described to trigger a defence system against late blight produced by the pathogen *Phytophthora infestans* [47]. Peptide no. 14 is obtained from translation initiation factor IF-2, a chloroplastic GTP- and mRNA-binding protein involved in protein synthesis [48]. The sequence of peptide no. 15 originates from a mitogen-activated kinase 9, which is an ATP-binding protein with kinase activity involved in plant intracellular signal transduction [49]. Peptide no. 17 contains 50% glycine and is produced from a homologue of vasodilator-stimulated phosphoprotein (VASP), which associates with actin proteins and regulates chemotaxis [50]. The sequence of peptide no. 18 derives from a hypothetically predicted protein with high homology to transcription factor bHLH143, a member of the basic/helix–loop–helix (bLHL) family of transcription factors, which bind to specific DNA sites and regulate many biological processes [51]. Peptide no. 19 seems to originate from a basic endochitinase-like protein. Arabidopsis endochitinases are involved in chitin degradation and protect plants from pathogen attack [52]. The sequences of peptides nos. 7 and 16 are part of hypothetically predicted proteins detected in the olive database with unknown functions as of yet. All of the proteins above are presumably present in the olive oil seed, mesocarp or epidermis and are subjected to degradation, either previously or during extraction of VOO, or perhaps originate from VOO proteins. 

The identification of olive peptides and proteins is a difficult task. The proteome of *Olea europaea* is, with a few exceptions, mostly uncharacterised, and the genomic and transcriptomic databases available contain computationally analysed records. As of December 2021, there are only 112 reviewed sequences in Swiss-Prot and 12,246 unreviewed TrEMBL sequences, i.e., translations of all coding sequences present in the EMBL/GenBank/DDBJ Nucleotide Sequence Databases as well as protein sequences extracted from the literature or submitted to UniProtKB/Swiss-Prot, that await revision. This is probably why the peptides that we found in olive oil have not been reported before and why only a few proteins have been identified as possible sources of peptides. 

### 2.3. Endogenous Peptides Identified by de Novo Sequencing in VOO

Eight novel peptides (not present in the olive database), with ALC % above 90, were confidently identified by Peaks de novo sequencing algorithm (Table 3). The peptides detected were short (six or seven amino acids) and rich in hydrophobic amino acids (>50%). The majority contained one or two histidines and/or cysteines in their sequences. The fragmentation spectra, total ion chromatogram and retention times of the de novo peptides reported in Table 3 are shown in Supplementary Materials Figure S1. The novel peptides identified by de novo sequencing with ALC% > 80 are shown in Supplementary Materials Table S3.

### 2.4. Predicted Activities of VOO Endogenous Peptides 

The predicted biological activities of the VOO endogenous peptides detected by Peaks DB and Peaks de novo sequencing algorithm are reported in Table 4 and Table 5, respectively. We used the BIOPEP database of bioactive peptides to investigate their biological activity because it uses previously published records to calculate a peptide’s theoretical activity, as well as the number of active motifs within each peptide sequence. 

According to BIOPEP, 24 peptides possessed ACE-inhibitory activity, and 26 peptides showed dipeptidyl peptidase (DPP)-III- and/or IV-inhibitory activity. As mentioned before, ACE inhibition is a target for the treatment of hypertension. DPP-III plays a role in blood pressure regulation, as well, by modulating angiotensin I and II [53]. Human DPP-IV is involved in blood glucose regulation, and its inhibition is used as a target for antidiabetic drugs [54]. Antioxidative activity was reported for eight peptide sequences. Other biological activities detected in the peptides were glucose uptake stimulation (see Table 4 nos. 2, 7, 8, 9), renin inhibitors, anti-inflammatory, antithrombotic, antiamnestic, opioid, stimulation of vasoactive substance release, alpha-glucosidase inhibitor, regulation of stomach mucosal activity and activator of ubiquitin-mediated proteolysis. New sources of ACE inhibitors and antioxidants are of interest for the food and pharmaceutical industries, so we focused on the study of those activities. Potential antioxidant activities of peptides are particularly interesting due to the implications they may have for the quality and stability of VOO. Other peptide activities, such as the inhibitors of DPP-III and IV, are also of great interest and could be the subject of further studies.

The capacity of peptides to produce systemic effects, such as inhibition of ACE, is conditioned by their bioavailability to reach blood and organs. Proteins and peptides are metabolised to single amino acids, but peptides containing two, three or more amino acids can be absorbed directly into the blood. This is determined by the proteolytic action of digestive enzymes. This aspect was investigated in our detected peptide sequences. The in silico-simulated proteolytic activity of pepsin, trypsin and chymotrypsin on the peptides identified by Peaks DB and by de novo sequencing produced different molecular species (Table 6). Digestive enzymes originated a number of di- and tri-peptides (QF, IF, RF, GF, VVL and SVY) with previously reported ACE- (or renin-) inhibitory activity [55], suggesting that some peptides would still be able to produce an inhibition of ACE after digestion in the gut. 

### 2.5. ACE-Inhibitory and Antioxidant Activity of Synthetic Peptides

We investigated the potential ACE-inhibitory and antioxidant activities by using synthetic peptides. Due to the high percentage of hydrophobic and neutral amino acids (>85%), water solubility was problematic for many peptide sequences, so they had to be excluded from the analysis. Out of the 27 sequences identified, seven Peaks DB peptides (nos. 1, 2, 5, 10, 14, 18 and 19), and two de novo sequencing peptides (nos. 1 and 2) were selected for in vitro studies using the selection criteria described in Section 2.5. We included peptide sequences containing one or more cysteine amino acids because thiol groups are very susceptible to oxidations, hence their potential antioxidant activity. This is the case of glutathione, a cysteine-containing tripeptide which is a principal cellular antioxidant. 

Peaks DB sequences VCGEAFGKA (no. 14), NALLCSNS (no. 18) CPANGFY (no. 19) and de novo identified sequences CCYSVY (no. 1) and DCHYFL (no. 2) showed strong ACE-inhibitory and antioxidant activities (Table 7). The C-terminal dipeptide KA present in sequence no. 14 has been reported to inhibit ACE with an IC_50_ value of 31.5 µM [56], which may explain the similar activity values detected by us. The ACE-inhibitory activity of NALLCSNS was remarkable and unexpected because no previous records of ACE-inhibitor peptides or di- or tripeptide fragments contained in the sequence were found that could explain the activity. Regarding CPANGFY (no. 19), the ACE-inhibitory values reported for C-terminal dipeptide FY (IC_50_ range 1.65–42.6 µM) [55,57] are in agreement with our results. Dipeptide GF, originated by the chymotrypsin digestion of the sequence (Table 6), also possesses ACE-inhibitory activity [58]. In addition, both FY and GF have been confirmed in vivo for antihypertensive activity. Dipeptide VY and tripeptide SVY present at the C-terminal end of de novo peptide no. 1 have been shown to possess strong ACE-inhibitory activities of the same order of magnitude of the well-known ACE-inhibitory (and antihypertensive) peptides IPP (IC_50_ 5 µM) and VPP (IC_50_ 9 µM) described in dairy [33,59]. Indeed, the IC_50_ values reported for VY and SVY are 7.1 µM and 8.1 µM, respectively [55,60]. The IC_50_ values measured by us for CYSVY are in agreement with the above results. Tripeptide SVY also seems to be produced by the proteolytic activity of chymotrypsin (Table 6). Regarding de novo peptide no. 2, C-terminal dipeptide FL has also been shown to produce inhibition of ACE with an IC_50_ value of 15.8 µM [55]. 

Antioxidant effects of plants are known mostly because of the radical scavenging properties of their polyphenolic, carotenoid and antioxidant vitamin contents. However, recent studies have shown that many proteins and peptides from plants possess strong antioxidant activity. This activity has been evaluated by using in vitro methods, such as the ABTS radical scavenging capacity, before being assessed in cellular systems or in vivo animal models [61,62,63]. Mechanisms proposed for the antioxidant activity of peptides include free radical scavenging and chelation of transition metals [64]; induction of antioxidant enzymes, such as catalase or superoxide dismutase [65]; induction of antioxidant molecules (e.g., glutathione), which protect cells from damage by reactive oxygen species [66,67]; and inhibition of oxidative enzymes, such as lipoxygenase, which catalyses oxidation of unsaturated fatty acids [14]. Antioxidant activity of peptides has been attributed to certain sequences of amino acids containing cysteine, methionine, lysine, histidine, tryptophan and tyrosine [68,69]. Hydrophobic peptides may also present advantages regarding their antioxidant activity because of their easier access to hydrophobic molecules, such as fatty acids [13]. 

VOO peptides VCGEAFGKA (no. 14), NALLCSNS (no. 18) and CPANGFY (no. 19), as well as de novo CCYSVY (no. 1) and DCHYFL (no. 2), all contained hydrophobic amino acids in the C-terminal position and also one or two antioxidant cysteines, which could explain their activity. The antioxidant activity reported for glutathione is 0.9 TEAC [70], so the antioxidant activity measured for the five peptides (0.8–3.2 TEAC) is quite strong. The presence of antioxidant peptides in VOO may positively affect the quality of the oil. Oxygen and light are inducers of lipid hydro peroxide radicals, which lead to oxidation of olive oil fatty acids. Polyphenols and alpha-tocopherols are able to quench oxygen free radicals and lipid peroxides, therefore protecting VOO from oxidation. The content of both antioxidant polyphenols and alpha-tocopherols are indicators of VOO quality [71]. Antioxidant peptides could also play a significant role in VOO quality. In addition, antioxidant peptides could act in cooperation with antioxidant polyphenols. For example, the antioxidant activity of peptides containing cysteine, once oxidised, may be recycled back to their reduced forms by polyphenols. In this regard, we have recently reported the presence of significant amounts of reduced glutathione (>5 mg/100 g) in mature olives, which may be recycled by olive polyphenols when glutathione is oxidised [72]. Although we could not detect glutathione in VOO (not shown), antioxidant peptides, such as the ones shown above, could have a similar antioxidant role to glutathione in olives. One limitation of this study is a lack of quantification of the peptides in VOO. Another limitation is that we only analysed the water-soluble peptide fraction of VOO, so it is quite likely that there are other peptide sequences with biological activity that have yet to be identified. At present, we are investigating the effect of the in vitro digestion of VOO peptides on their ACE-inhibitory activity. The antihypertensive and antioxidant effects of VOO endogenous peptides are also being tested in vivo by using the spontaneously hypertensive rat model of hypertension. This animal model allows for investigation of the long- and short-term effects of bioactive peptides on blood pressure, as well as use of the biomarkers of oxidation to explore antioxidant effects. Those results should corroborate the antihypertensive and antioxidant effects in vivo of VOO peptides.

## 3. Materials and Methods

### 3.1. Plant Material and Extraction of Olive Oil 

Olive fruits (*Olea europea* L. variety Picual) were hand-picked from healthy olive trees in the province of Granada (Spain). The olives used for extraction of oil were collected at the beginning of the maturation stage with a ripening index 5, according to the scale described in [73], when olives have black skin and white pulp. Extraction of the olive oil was carried out using a laboratory scale olive oil extraction system (Abencor, MC2 Ingeniería y Sistemas SL, Sevilla, Spain), as previously described by [74]. Filtration of the obtained olive oil was always avoided.

### 3.2. Extraction and Fractionation of VOO Peptides 

Olive oil peptides were extracted from VOO, as described in [11]. In short, peptides were precipitated with a mixture of cold acetone:hexane (1:1) and the mixture was then centrifuged at 10,000 g for 15 min at 4 °C. The supernatant was discarded, and the precipitates were pooled and desiccated. Milli-Q water was used to solubilise the peptides with the aid of sonication, followed by centrifugation as above. The solubilised peptides were then subjected to gel filtration chromatography using fast protein liquid chromatography (FPLC) as described in [35]. The fractions containing molecular masses ranging from 1600 to 700 were selected. The fractions were pooled, then dried by using a rotary evaporator and stored at −80 °C until further analysis. 

### 3.3. LC-Orbitrap MS/MS Analysis of Peptide Fractions

The peptide fractions extracted from VOO described above were analysed by nanoscale liquid chromatography-Orbitrap coupled with tandem mass spectrometry (nanoLC-Orbitap-MS/MS) and de novo sequencing. For this, the dried extract described above was dissolved with 1 mL of Milli-Q water, and then it was diluted 10-fold with 0.1% formic acid prepared in Milli-Q water. Using nanoLC-Orbitrap MS/MS in duplicates, 5 μL of samples (containing 0.3 μg of proteins) were analysed. The peptides were separated onto a C-18 reversed phase nano-column (75 μm I.D.; 15 cm length; 3 μm particle diameter, Nikkyo Technos Co. LTD, Japan) coupled to a trap nano-column (100 μm ID; 2 cm length; 5 μm particle diameter, Thermo Fisher Scientific, Waltham, Massachussets, USA). The chromatographic separation was performed with 0.1% formic acid in Milli-Q water (phase A) and 0.1% formic acid in acetonitrile (phase B), using the following gradient: 0 to 5% B in 2 min, 5 to 35% of B in 30 min, 35 to 75% B in 20 min and 75 to 95% B in 5 min, maintained for 10 min. A flow rate of 300 nl/min was used to elute peptides for real time ionisation and peptide fragmentation on an LTQ-Orbitrap Velos Pro mass spectrometer (Thermo Fisher). An enhanced Fourier-transform MS/MS-resolution spectrum (resolution = 30,000 full width at half maximum) was obtained, followed by a data dependent MS/MS scan. The data dependent MS/MS event consists of collision-induced dissociation fragmentation (35% normalized collision energy) and ion trap-MS/MS acquisition from the most intense ten parent ions with a charge state rejection of +1 (Z = 1) and dynamic exclusion of 0.5 min, which is typically used for peptide identification. Interferences with other possible organic compounds were reduced by selecting only precursor ions with a minimum charge of +2 (Z = 2) or higher. 

### 3.4. Peptide and Protein Identification by de Novo Sequencing and Database Search 

Peaks X Pro (Bioinformatics Solutions Inc., Waterloo, ON, Canada) software was used for the treatment of the raw mass spectrometry data obtained by the nanoLC-Orbitap-MS/MS analysis. De novo sequencing and Peaks database searching-based protein identification (Peaks DB) were used to identify the most likely peptide sequence that matched the resulting spectra obtained from the LC-MS/MS analysis [38]. The parameters used for the analysis include parent mass error tolerance: 10.0 ppm; fragment mass error tolerance: 0.5 Da; precursor mass search type: monoisotopic; enzyme: none; variable modifications: oxidation of methionine. De novo peptide sequencing results obtained by Peaks are confirmed by local confidence scores at the amino acid level. The local confidence score ranges from 0% to 99%, showing how confidently the algorithm considers a particular amino acid in the sequence as the correct assignment. The results were filtered by using the average local confidence (ALC) parameter, which is the average of the local confidence score of all the amino acids in the sequence. An ALC value above 90% (over this value, the sequence prediction is great, according to Peaks) was used to filter the results obtained from de novo sequencing analysis. 

Database searches were carried out with Peaks DB algorithm, using the available *Olea europaea* (cv. Farga) genome database [75], containing 55,595 protein-coding genes, which can be downloaded at [76]. Peptide-spectrum matches were filtered by peptide −10lgP score. This parameter is calculated for every peptide-precursor spectrum match reported by Peaks DB and indicates the statistical significance of the peptide-precursor spectrum match (the quality of the match). The score is derived from the *p*-value, defined by Peaks as the probability that a false identification in the database search achieves the same, or better, matching score. For example, a −10 lgP score of 20 (suggested by Peaks as a starting value to filter peptide-spectrum matches) is equivalent to a *p*-value of 0.01, which indicates that the probability of the peptide amino acid sequence being false is ≤ 1%. According to Peaks, a −10 lgP score > 30 indicates a peptide sequence identified with high confidence [77,78]. In our study, we used a −10 lgP score threshold of 30 (*p* = 0.001) to filter for peptide-spectrum matches. Peptide identifications were accepted if they appeared in at least two individual samples. Identified proteins were also filtered using a −10 lgP score of 30. The protein score is calculated as the weighted sum of the −10 lgP scores of the protein’s supporting peptides. Relative abundance of the peptides was calculated by Peaks based on peptide features detected from LC-MS/MS data by integrating the area under the curve (label-free quantification). Protein abundance was calculated by the addition of areas obtained from the supporting peptides. The protein sequences retrieved from the database search were subjected to NCBI BLAST-P search [79] to look for significant alignments and similarities with other previously identified proteins present in their databases (including *Olea europaea*). Protein identification matches of the highest quality, with an identity percentage of 100% and a cover percentage >95%, were reported. 

### 3.5. Biological Activity Prediction of VOO Peptides and Peptide Selection 

Biological activities of the peptides obtained by de novo sequencing and database search were explored by using the BIOPEP-UWM database of bioactive peptides [80]. This database contains referenced data from 4399 bioactive peptides (as of December 2021), including 1064 inhibitors of ACE and 756 antioxidant peptides [81]. Potential biological activity of the peptide sequence is calculated by the database by using parameters A and B. Parameter A is the number of sequences with the previously reported activity divided by total number of amino acid residues (N) of the peptide sequence. Parameter B is the potential biological activity of the peptide (µM^−1^) calculated using published data of the activity of sequence motif(s) present in the peptide [82]. The action of digestive enzymes on endogenous peptides identified by Peaks DB and de novo sequencing was studied in silico. Biological activity of the peptide fragments originated from the proteolytic activity of pepsin, trypsin and chymotrypsin was investigated by using the BIOPEP database. A selection of potentially active peptides was carried out for subsequent in vitro studies. The criteria we used for the peptide selection were: water solubility (% of hydrophobicity), ACE-inhibitor and antioxidant activity predicted by BIOPEP (confirmed by activity of the proteolytic fragments originated by digestive enzymes), peptide relative abundance, the presence of one or more hydrophobic or positively charged amino acids within the three amino acids closest to the C-terminal position [83], and the presence of one or more cysteine amino acids in the sequence.

### 3.6. Peptide Synthesis 

Selected peptides were synthesised by CASLO ApS (Scion Denmark Technical University, Lyngby, Denmark). The purity and molecular mass of the synthesised peptides were certified by analytical HPLC and mass spectrometry (MALDI-TOF, not shown). The purity of the synthetic peptides was 97.1% on average (range 91.9–99.3%). All peptide matches were approved by the supplier. The peptides were used to investigate their antioxidant and ACE-inhibitory activities. 

### 3.7. Peptide Concentration Determination 

The total peptide concentrations in the olive oil extracts were determined by fluorescence using a protein quantification kit (FluoroProfile, Sigma-Aldrich, Saint Louis, Missouri, USA). BSA was used as standard solution. Fluorometric quantification was carried out to 530 nm and 630 nm as excitation and emission wavelengths, respectively.

### 3.8. Determination of Angiotensin-Converting Enzyme Inhibitory Activity

The ACE-inhibitory activity of the synthetic peptides was determined according to the method described in [84], with some modifications. This assay is based on the ability of ACE to hydrolyse the substrate o-aminobenzoylglycyl-p-nitrophenylalanyl-proline (Abz-Gly-Phe-(NO_2_)-Pro, Bachem Feinchemikalien, Bubendorf, Switzerland), producing the fluorescent product o-aminobenzoylglycine (Abz-Gly). Reagents used include: buffer A: 150 mM Tris-HCl buffer (pH 8.3), with 0.1 µM ZnCl_2_; buffer B: 150 mM de Tris-HCl buffer (pH 8.4), with 1125 mM NaCl; ACE solution (stock): rabbit-lung ACE (E.C.3.4.15.1., Sigma-Aldrich), dissolved in buffer A containing 50% glycerol to make an enzyme concentration of 1 U/mL. This solution was diluted 1/20 with buffer A to obtain the working solution (50 mU/mL), which was prepared fresh every day to conduct the experiment. The substrate solution (Abz-Gly-Phe(NO_2_)-Pro) was dissolved in buffer B to a final concentration of 0.45 mM. This solution was also prepared every day before its use and was protected from light and kept at 4 °C. The assay was carried out using a fluorescence technique. Black polystyrene plates of 96 wells (Thermo Scientific, Waltham, Massachussets, USA) were used. The wells contained the following reaction solutions: control = 50 μL of Milli-Q water and 50 μL of ACE solution; blank = 50 μL of Milli-Q water and 50 μL of buffer A; sample = 50 μL of sample and 50 μL of ACE solution; sample blank = 50 μL of sample and 50 μL of buffer A. The enzymatic reaction was initiated by adding 200 μL (final volume in each well 300 μL) of substrate solution, and the plate was immediately mixed and incubated at 37 °C in a VICTORX5 fluorometer (PerkinElmer, USA). The fluorescence generated was measured after 30 min using 355 and 420 nm as excitation and emission wavelengths, respectively. The ACE-inhibitory activity of each sample was determined in triplicate. The ACE-inhibitory activity was calculated using the following formula:

$$\mathrm{ACE}\;\mathrm{inhibitory}\;\mathrm{activity}\;\left(\%\right)=\frac{\left(\mathrm{FC}-\mathrm{FB}\right)-\left(\mathrm{FS}-\mathrm{FBs}\right)}{\mathrm{FC}-\mathrm{FB}}\;\times \;100$$

where FC (control) is the fluorescence emitted after the action of ACE on the substrate, without inhibitor (i.e., sample); FB (blank) is the fluorescence emitted by the substrate; FS (sample) is the fluorescence emitted after the action of ACE on the substrate, with inhibitor sample; and FBs (blank sample) are the fluorescences emitted by the substrate and the sample.

The ACE-inhibitory activity is expressed as IC_50_, which is the concentration of inhibitor required to inhibit the activity of ACE by 50%.

### 3.9. Determination of the Antioxidant Activity 

The antioxidant capacity of the synthetic peptides was assessed by using the ABTS [2,2´-azino-bis(3-ethylbenzothiazoline-6-sulphonic acid)] radical cation decolorization (scavenging) assay, as described in [85]. Calibration curves of the antioxidant Trolox (0–1 mM) were used as standards. All ABTS assays were carried out at least three times and in triplicate at each concentration of the Trolox standards and peptide samples. The values of antioxidant capacity of the synthetic peptides were expressed in TEAC (Trolox Equivalent Antioxidant Capacity). TEAC is defined as the millimolar concentration of a Trolox solution with equivalent antioxidant potential to a 1 mM concentration of the compound under investigation [70]. 

### 3.10. Statistical Analysis

Data are expressed as means ± standard error of the mean (SEM). Differences of *p* < 0.05 between the groups were considered significant. SPSS statistical software version 23.0 was used for the statistical analysis (SPSS, Chicago, IL, USA).

## 4. Conclusions

We identified 19 new peptides, as well as their proteins sources, and eight de novo sequencing peptides in a water-soluble extract of VOO. The peptide sequences were predicted to possess different types of bioactivity. Synthetic peptides VCGEAFGKA, NALLCSNS, CPANGFY, CCYSVY and DCHYFL possessed strong ACE-inhibitory and antioxidant activities in vitro. More research is needed to clarify the potential role of antioxidant peptides in VOO quality and their biological effects in humans. 

## Figures and Tables

**Table 1:** Endogenous peptides identified in virgin olive oil using Peaks database (DB) algorithm: −10 lgP score, statistical significance of the peptide-precursor spectrum match; N, number of amino acids; m/z, precursor mass-to-charge ratio; mass, peptide mass in Da; RT, retention time; ppm, precursor mass error; area, peptide abundance shown as area under curve of detected peptide; protein source, best-matched protein identified by BLAST-P search; accession, the accession number of the highest-scoring protein containing this peptide. Oxidised methionine is indicated by M (+15.99). ND, not detected.

No.	Sequence	−10 lgP	N	m/z	Mass	RT	ppm	Area	Protein Source (BLASTP)	Accession
1	LDTANEMNQLDLQFR	93.56	15	904.4365	1806.8571	34.18	8.3	1.0 × 10^6^	11S globulin subunit beta-like	OE9A085162P3: OE9A085162P1: OE9A085162P2
2	VVLQDTSNNVNQLD	77.73	14	779.8878	1557.7634	24.96	5.9	3.44 × 10^5^	legumin A-like	OE9A032471P1: OE9A042009P1
3	VVLQDTSNNVNQLDDIPRRFFLA	63.26	23	892.1344	2673.3875	48.98	5.2	ND	legumin A-like	OE9A032471P1: OE9A042009P1
4	AVVPIWLQPDTPAR	57.79	14	781.9397	1561.8616	38.09	9.6	4.39 × 10^5^	apyrase-like	OE9A001718P2: OE9A001718P1
5	IFSGGESSGQPR	57.53	12	611.2961	1220.5785	22.47	6.8	1.24 × 10^5^	legumin A-like	OE9A032471P1: OE9A042009P1
6	DTSNNVNQLDDIPRR	46.89	15	586.2892	1755.8500	26.25	5.1	1.54 × 10^5^	legumin A-like	OE9A032471P1: OE9A042009P1
7	NCSTSIISG	39.01	9	441.2062	880.3961	35.88	9.5	3.49 × 10^7^	hypothetical predicted protein	OE9A087501P1
8	VHVFRFDQNQDLLPIGN	34.71	17	671.3506	2011.0275	48.03	8.8	1.53 × 10^4^	apyrase-like	OE9A001718P2: OE9A001718P1
9	LLLGAGCM(+15.99)	34.51	8	397.1985	792.3874	33.76	1.4	1.24 × 10^7^	zinc transporter 5-like	OE9A117792P1
10	QDTSNNVNQLDDIPRR	33.36	16	628.9771	1883.9086	26.36	8.0	2.06 × 10^6^	legumin A-like	OE9A032471P1: OE9A042009P1
11	FSASTEGS	32.74	8	393.1665	784.3239	25.27	0.5	9.63 × 10^5^	probable L-type lectin domain containing receptor kinase S.7	OE9A013295P1: OE9A042038P1: OE9A062197P1
12	INTISGR	31.90	7	380.7197	759.4239	20.53	8.6	1.38 × 10^5^	legumin A-like	OE9A032471P1: OE9A042009P1
13	LDGNSSAR	31.66	8	410.1964	818.3882	18.63	−4.9	1.41 × 10^5^	late blight resistance protein homolog R1A-10	OE9A106456P1
14	VCGEAFGKA	31.36	9	441.2060	880.4113	33.38	−8.2	4.18 × 10^6^	translation initiation factor IF-2, chloroplastic	OE9A058866P1
15	KGGGGGSGSAGGGGS	31.14	15	525.2275	1048.4534	35.95	−4.9	4.01 × 10^6^	mitogen-activated kinase 9-like, partial	OE9A057915P1
16	SGPGNHEQ	30.94	8	413.1722	824.3413	30.38	−6.5	ND	hypothetical predicted protein	OE9A054657P1: OE9A104108P1
17	LGGGGSSGGAAC	30.30	12	447.1878	892.3708	20.67	−3.4	4.31 × 10^5^	VASP homolog (vasodilator-stimulated phosphoprotein)	OE9A026632P1
18	NALLCSNS	30.09	8	411.1950	820.3749	27.63	8.1	2.25 × 10^5^	transcription factor bHLH143-like	OE9A100476P1
19	CPANGFY	29.54	7	386.1598	770.3057	26.19	6.7	ND	basic endochitinase-like	OE9A087887P1

**Table 2:** Proteins identified as sources of endogenous peptides detected in virgin olive oil: protein −10 lgP score, sum of −10 lgP scores of a protein’s supporting peptides; peptides, number of high-confidence supporting peptides; cover (%), percentage of the protein sequence covered by supporting peptides; area, protein relative abundance; protein identification, definitions of best-matched proteins identified by BLAST-P search; E-value (score), number of expected hits of similar quality that could be found randomly; protein description, access to protein report.

Protein Accession	−10 lgP	Peptides	Cover (%)	Average Mass (Da)	Area	Protein Identification (BLASTP)	E-Value	% Identity	Protein Description
OE9A032471P1	155.75	6	9	51,572	2.82 × 10^6^	legumin A-like	0.0	100	CAA3011204.1
OE9A042009P1	155.75	6	9	51,725	2.82 × 10^6^	legumin A-like	0.0	100	ID: CAA3019014.1
OE9A085162P3	93.56	1	4	40,777	1.08 × 10^6^	11S globulin subunit beta-like	0.0	100	XP_022872671.1
OE9A085162P1	93.56	1	4	44,093	1.08 × 10^6^	11S globulin subunit beta-like	0.0	100	CAA2975576.1
OE9A085162P2	93.56	1	3	55,757	1.08 × 10^6^	11S globulin subunit beta-like	0.0	100	XP_022872671.1
OE9A001718P2	75.15	2	7	47,253	4.54 × 10^5^	apyrase-like	0.0	100	CAA3020286.1
OE9A001718P1	75.15	2	7	49,955	4.54 × 10^5^	apyrase-like	0.0	100	CAA3020285.1
OE9A057915P1	43.19	1	7	21,805	4.01 × 10^6^	mitogen-activated kinase 9-like	6 × 10^−86^	100	CAA3032954.1
OE9A117792P1	43.16	1	2	38,475	6.11 × 10^6^	zinc transporter 5-like	0.0	100	CAA3032762.1
OE9A087501P1	39.01	1	4	27,647	3.49 × 10^7^	hypothetical predicted protein	1 × 10^−160^	100	CAA2984267.1
OE9A013295P1	32.74	1	1	72,709	9.63 × 10^5^	probable L-type lectin domain containing receptor kinase S.7	0.0	100	CAA2995736.1
OE9A042038P1	32.74	1	1	75,341	9.63 × 10^5^	probable L-type lectin domain containing receptor kinase S.7	0.0	100	XP_022879160.1
OE9A062197P1	32.74	1	1	75,780	9.63 × 10^5^	probable L-type lectin domain containing receptor kinase S.7	0.0	100	CAA2978283.1
OE9A106456P1	31.66	1	1	86,941	7.40 × 10^4^	late blight resistance protein homolog R1A-10	0.0	100	CAA2992824.1
OE9A058866P1	31.36	1	1	108,486	4.18 × 10^6^	translation initiation factor IF-2, chloroplastic	0.0	100	CAA3007316.1
OE9A054657P1	30.94	1	3	30,439	0	hypothetical predicted protein	4 × 10^−138^	100	CAA3010733.1
OE9A104108P1	30.94	1	2	41,131	0	hypothetical predicted protein	2 × 10^−172^	100	CAA2977756.1
OE9A026632P1	30.3	1	4	30,682	4.31 × 10^5^	VASP homolog (vasodilator-stimulated phosphoprotein)	2 × 10^−136^	100	CAA2968008.1
OE9A100476P1	30.09	1	2	38,275	2.25 × 10^5^	hypothetical predicted protein (transcription factor bHLH143-like)	0.0	100	CAA2983039.1
OE9A087887P1	29.54	1	3	28,540	0	basic endochitinase-like	0.0	100	CAA2941635.1

**Table 3:** Endogenous peptides identified in virgin olive oil by de novo sequencing: ALC, average local confidence; N, number of amino acids; m/z, precursor mass-to-charge ratio; mass, peptide mass in Da; RT, retention time; area, peptide abundance shown as area under the curve of the detected peptide; ppm, precursor mass error; local confidence, local confidence score for each amino acid of the peptide identified by de novo sequencing. Oxidised methionine is indicated by a pair of parentheses enclosing the modification mass (+15.99). ND, not detected.

No.	Peptide	ALC (%)	N	m/z	Mass	RT	Area	ppm	Local Confidence (%)
1 de novo	CCYSVY	95	6	369.1365	736.256	24.36	5.77 × 10^5^	3.2	92 97 99 93 92 98
2 de novo	DCHYFL	94	6	399.1715	796.3214	25.13	6.41 × 10^4^	8.9	92 98 98 98 90 93
3 de novo	LYPFAH	94	6	374.1952	746.3751	19.07	3.59 × 10^5^	1	99 99 99 85 89 93
4 de novo	SVSKPGW	92	7	380.7006	759.3915	14.03	1.85 × 10^5^	−6.5	93 97 99 99 89 78 93
5 de novo	LHTVVH	91	6	353.2084	704.397	26.06	8.67 × 10^4^	7.6	99 93 93 92 85 87
6 de novo	NKLCCEH	91	7	423.6815	845.3524	29.15	ND	−4.7	84 95 98 94 86 94 89
7 de novo	DHHEEL	91	6	390.1661	778.3246	36.35	7.71 × 10^4^	−8.9	89 91 97 91 92 85
8 de novo	LPCAAHR	90	7	384.2062	766.3908	19.09	2.70 × 10^5^	9.2	98 79 79 89 97 96 95

**Table 4:** Predicted activity of VOO endogenous peptides identified by Peaks DB using BIOPEP-UWM database of bioactive peptides: ACE, angiotensin-converting enzyme; DPP III, dipeptidyl peptidase-III; DPP IV, dipeptidyl peptidase-IV; A, ratio of number of sequences with predicted activity to number of amino acids; B, potential biological activity of the peptide (µM^−1^) theoretically calculated from previous bioactive referenced data. A and B values obtained from BIOPEP-UWM database.

No.	Peptide Sequence	Predicted Activity	No. of Sequences with Activity	A	B
1	LDTANEMNQLDLQFR	ACE inhibitor	3	0.2000	7.24 × 10^−5^
		DPP IV inhibitor	7	0.4667	
		DPP III inhibitor	1	0.0667	
		Renin inhibitor	1	0.0667	
2	VVLQDTSNNVNQLD	ACE inhibitor	2	0.1429	
		Glucose uptake stimulation	1	0.0714	
		DPP IV inhibitor	9	0.6429	9.65 × 10^−4^
3	VVLQDTSNNVNQLDDIPRRFFLA	ACE inhibitor	9	0.3913	1.36 × 10^−2^
		Activator of ubiquitin-mediated proteolysis	1	0.0435	
		DPP IV inhibitor	10	0.2174	
		DPP III inhibitor	5	0.6087	8.62 × 10^−4^
		Stimulation	1	0.0435	
4	AVVPIWLQPDTPAR	ACE inhibitor	10	0.7143	5.69 × 10^−2^
		DPP IV inhibitor	10	0.7143	1.80 × 10^−3^
5	IFSGGESSGQPR	ACE inhibitor	9	0.7500	4.63 × 10^−2^
		Antioxidant	1	0.0833	
		DPP III inhibitor	2	0.1667	
		DPP IV inhibitor	4	0.3333	
		Neuropeptide	1	0.0833	
6	DTSNNVNQLDDIPRR	ACE inhibitor	3	0.2000	1.70 × 10^−2^
		DPP IV inhibitor	8	0.1333	
		DPP III inhibitor	2	0.5333	1.62 × 10^−4^
7	NCSTSIISG	ACE inhibitor	2	0.2222	2.75 × 10^−2^
		DPP IV inhibitor	3	0.3333	
		Glucose uptake stimulation	1	0.1111	
8	VHVFRFDQNQDLLPIGN	ACE inhibitor	5	0.2941	8.20 × 10^−3^
		Glucose uptake stimulation	1	0.0588	
		DPP IV inhibitor	11	0.6471	2.48 × 10^−5^
		DPP III inhibitor	2	0.1176	
9	LLLGAGCM(+15.99)	ACE inhibitor	3	0.3750	1.26 × 10^−4^
		Glucose uptake stimulation	2		
		Stimulation of vasoactive substance release	1	0.3750	
		DPP IV inhibitor	3	0.5000	
10	QDTSNNVNQLDDIPRR	ACE inhibitor	3	0.1875	1.59 × 10^−2^
		DPP IV inhibitor	9	0.5625	1.52 × 10^−4^
		DPP III inhibitor	2	0.1250	
11	FSASTEGS	ACE inhibitor	4	0.5000	3.11 × 10^−2^
		DPP IV inhibitor	3	0.3750	
12	INTISGR	ACE inhibitor	2	0.2857	6.14 × 10^−5^
		DPP IV inhibitor	3	0.4286	
13	LDGNSSAR	ACE inhibitor	2	0.2500	1.14 × 10^−2^
14	VCGEAFGKA	ACE inhibitor	7	0.7778	4.80 × 10^−3^
		Alpha-glucosidase inhibitor	1	0.1111	6.53 × 10^−6^
		DPP IV inhibitor	3	0.3333	1.78 × 10^−5^
		DPP III inhibitor	2	0.2222	
15	KGGGGGSGSAGGGGS	ACE inhibitor	13	0.8667	1.72 × 10^−4^
		DPP IV inhibitor	9	0.6000	
16	SGPGNHEQ	ACE inhibitor	4	0.5000	2.10 × 10^−3^
		Antiamnestic	2	0.2500	
		Antithrombotic	2	0.2500	
		Regulation of stomach mucosal activity	2	0.2500	
		DPP IV inhibitor	4	0.5000	1.80 × 10^−3^
17	LGGGGSSGGAAC	ACE inhibitor	9	0.7500	2.63 × 10^−4^
		Antioxidant	1	0.0833	
		DPP IV inhibitor	6	0.5000	8.87 × 10^−6^
18	NALLCSNS	Glucose uptake stimulation	1	0.1250	
		DPP IV inhibitor	3	0.3750	1.42 × 10^−6^
19	CPANGFY	ACE inhibitor	3	0.4286	5.90 × 10^−3^
		DPP IV inhibitor	3	0.4286	
		DPP III inhibitor	1	0.1429	

**Table 5:** Predicted activity of endogenous peptides identified in virgin olive oil by de novo sequencing using BIOPEP-UWM database of bioactive peptides: ACE, angiotensin converting enzyme; DPP III, dipeptidyl peptidase-III; DPP IV, dipeptidyl peptidase-IV; A, ratio of number of sequences with predicted activity to number of amino acids; B, potential biological activity of the peptide (µM^−1^) theoretically calculated from previous bioactive referenced data. A and B values obtained from BIOPEP-UWM database.

No.	Peptide	ALC (%)	Predicted Activity	No. Seq. with Activity	A	B
1 de novo	CCYSVY	95	ACE inhibitor	2	0.3333	4.39 × 10^−2^
			Antioxidant	1	0.1667	
			DPP III inhibitor	3	0.1667	
			DPP IV inhibitor	1	0.5000	
2 de novo	DCHYFL	94	ACE inhibitor	1	0.1667	6.38 × 10^−3^
			Anti-inflammatory	1	0.1667	
			DPP III inhibitor	3	0.3333	
			DPP IV inhibitor	2	0.5000	4.17 × 10^−4^
3 de novo	LYPFAH	94	ACE inhibitor	4	0.6667	3.47 × 10^−4^
			Alpha-glucosidase inhibitor	1	0.1667	9.92 × 10^−5^
			Antioxidant	2	0.3333	
			DPP III inhibitor	2	0.3333	
			DPP IV inhibitor	4	0.6667	5.25 × 10^−5^
			Opioid	1	0.1667	
			Renin inhibitor	1	0.1667	
4 de novo	SVSKPGW	92	ACE inhibitor	3	0.4286	1.12 × 10^−2^
			Antiamnestic	1	0.1429	
			Antioxidant	1	0.1429	
			Antithrombotic	1	0.1429	
			Regulation of stomach activity	1	0.1429	
			DPP IV inhibitor	6	0.8571	5.62 × 10^−5^
5 de novo	LHTVVH	91	Antioxidant	2	0.3333	
			DPP IV inhibitor	5	0.8333	
6 de novo	NKLCCEH	91	ACE inhibitor	3	0.4286	4.64 × 10^−3^
			DPP IV inhibitor	1	0.1429	
7 de novo	DHHEEL	91	Antioxidative	3	0.5000	
			Stimulation of vasoactive substance release	1	0.1667	
			DPP IV inhibitor	2	0.3333	
8 de novo	LPCAAHR	90	ACE inhibitor	2	0.2857	2.30 × 10^−4^
			Antioxidant	1	0.1429	
			DPP IV inhibitor	4	0.5714	7.54 × 10^−5^

**Table 6:** Molecular species (predicted) produced by the proteolytic activity of main digestive enzymes acting on peptides identified by Peaks DB and by PEAKS *de novo* sequencing: ACE, angiotensin converting enzyme; DPP, dipeptidyl peptidase. Peptide fragments with previously reported bioactivity are shown in red.

No.	Peptide Sequence	Pepsin	Trypsin	Chymotrypsin
1	LDTANEMNQLDLQFR	L-DTANEMNQL-DL-QF-R		L-DTAN-EM-N-QL-DL-QF-R
		QF: renin inhibitor,		QF, renin inhibitor,
		QF, DPP IV, inhibitor		QF, QL: DPP IV, inhibitor
2	VVLQDTSNNVNQLD	VVL-QDTSNNVNQL-D	VVL-QDTSNNVNQL-D	VVL-QDTSN-N-VN-QL-D
		VVL: ACE inhibitor	VVL: ACE inhibitor	VVL: ACE inhibitor
				QL, VN: DPP IV, inhibitor
3	VVLQDTSNNVNQLDDIPRRFFLA	VVL-QDTSNNVNQL-DDIPRRF-F-L-A		VVL-QDTSN-N-VN-QL-DDIPRRF-F-L-A
		VVL: ACE inhibitor		VVL: ACE inhibitor
				QL, VN: DPP IV, inhibitor
4	AVVPIWLQPDTPAR			
5	IFSGGESSGQPR	IF-SGGESSGQPR		IF-SGGESSGQPR
		IF: ACE inhibitor		IF: ACE inhibitor
6	DTSNNVNQLDDIPRR			DTSN-N-VN-QL-DDIPRR
				QL, VN: DPP IV, inhibitor
7	NCSTSIISG			
8	VHVFRFDQNQDLLPIGN	VHVF-RF-DQNQDL-L-PIGN		VHVF-RF-DQNQDL-L-PIGN
		RF: ACE inhibitor		RF: ACE inhibitor
		RF: DPP-III inhibitor		RF: DPP-III inhibitor
9	LLLGAGCM(+15.99)			
10	QDTSNNVNQLDDIPRR			QDTSN-N-VN-QL-DDIPRR
				QL, VN: DPP IV, inhibitor
11	FSASTEGS			F-SASTEGS
12	INTISGR			IN-TISGR
				IN: DPP-IV inhibitor
13	LDGNSSAR			
14	VCGEAFGKA			
15	KGGGGGSGSAGGGGS			
16	SGPGNHEQ			
17	LGGGGSSGGAAC			
18	NALLCSNS			N-AL-L-CSN-S
				AL: DPP-IV inhibitor
19	CPANGFY			CPAN-GF-Y
				GF: ACE inhibitor
				GF: DPP IV, inhibitor
				GF: DPP III inhibitor
1 de novo	CCYSVY			CCY-SVY
				SVY: ACE inhibitor
2 de novo	DCHYFL			
3 de novo	LYPFAH			L-YPF-AH
				YPF: Opioid
4 de novo	SVSKPGW			
5 de novo	LHTVVH			
6 de novo	NKLCCEH			
7 de novo	DHHEEL			
8 de novo	LPCAAHR			

**Table 7:** ACE-inhibitory activity and ABTS radical antioxidant capacity of a selection of synthetic peptides detected in virgin olive oil. Peptide nos. 1, 2, 5, 10, 14, 18 and 19 were identified by Peaks DB algorithm; peptide nos.1 de novo and 2 de novo were identified by Peaks de novo sequencing algorithm: ACEi activity, angiotensin-converting enzyme inhibitory activity; IC_50_ (µM), peptide concentration necessary to inhibit 50% of ACE activity, expressed in µM. Antioxidant activity is referred to Trolox and expressed as TEAC (Trolox Equivalent Antioxidant Capacity). Data are means ± SD of three different experiments. ND, not detected.

No.	Peptide Sequence	ACEi Activity IC_50_ (µM) ± SD	TEAC ± SD
1	LDTANEMNQLDLQFR	ND	ND
2	VVLQDTSNNVNQLD	ND	ND
5	IFSGGESSGQPR	ND	ND
10	QDTSNNVNQLDDIPRR	ND	ND
14	VCGEAFGKA	57.2 ± 4.31	0.79 ± 0.02
18	NALLCSNS	0.98 ± 0.04	0.95 ± 0.03
19	CPANGFY	3.76 ± 0.39	1.53± 0.14
1 de novo	CCYSVY	25.6 ± 0.41	3.20 ± 0.19
2 de novo	DCHYFL	41.5 ± 2.09	2.36 ± 0.02

## Data Availability

The data presented in this study are available on request made to the corresponding author.

## Supplementary Materials

The following supporting information can be downloaded at: https://www.mdpi.com/article/10.3390/ijms23031712/s1.
