# Supplementary material for: Characterisation of Endogenous Peptides Present in Virgin Olive Oil
Source: Int J Mol Sci. 2022 Feb 2;23(3):1712. doi: 10.3390/ijms23031712 (PMC8836281; doi:10.3390/ijms23031712)
Supplement: Supplementary file 1 [file ijms-23-01712-s001.zip › 1. Supplemental material Figure S1.pdf]

Supplemental material Figure S1. Fragmentation spectra, total ion chromatogram and amino acid sequence of peptides identified in VOO by Peaks by *de novo* sequencing algorithm. Fragment ion tables for b and y ions (monoisotopic masses) are included.

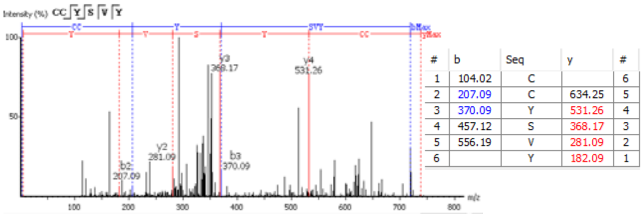

#	b	Seq	y	#
1	104.02	C		6
2	207.09	C	634.25	5
3	370.09	Y	531.26	4
4	457.12	S	368.17	3
5	556.19	V	281.09	2
6		Y	182.09	1

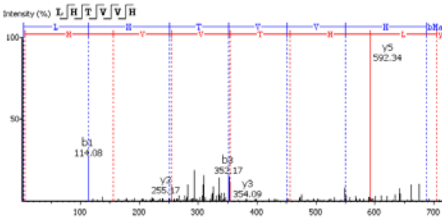

#	b	Seq	y	#
1	114.08	L		6
2	251.17	H	592.34	5
3	352.17	T	455.01	4
4	451.34	V	354.09	3
5	550.34	V	255.17	2
6		H	156.17	1

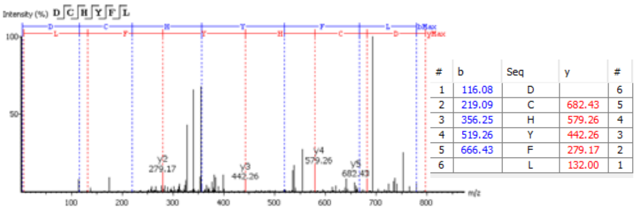

#	b	Seq	y	#
1	116.08	D		6
2	219.09	C	682.43	5
3	356.25	H	579.26	4
4	519.26	Y	442.26	3
5	666.43	F	279.17	2
6		L	132.00	1

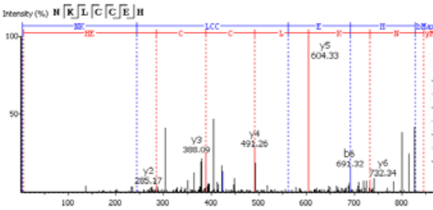

#	b	Seq	y	#
1	115.05	N		7
2	243.09	K	732.34	6
3	356.23	L	604.33	5
4	459.24	C	491.26	4
5	562.34	C	388.09	3
6	691.32	E	285.17	2
7		H	156.08	1

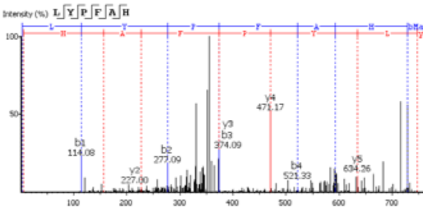

#	b	Seq	y	#
1	114.08	L		6
2	277.09	Y	634.26	5
3	374.09	P	471.17	4
4	521.33	F	374.09	3
5	592.26	A	227.00	2
6		H	156.08	1

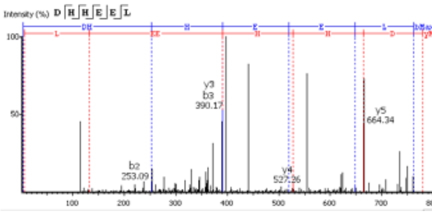

#	b	Seq	y	#
1	116.03	D		6
2	253.09	H	664.34	5
3	390.17	H	527.26	4
4	519.26	E	390.17	3
5	648.18	E	261.14	2
6		L	132.08	1

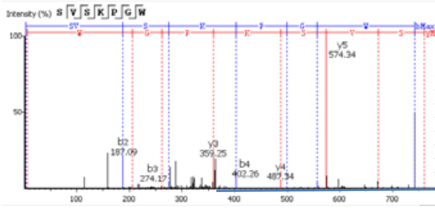

#	b	Seq	y	#
1	88.04	S		7
2	187.09	V	673.59	6
3	274.17	S	574.34	5
4	402.26	K	487.34	4
5	499.26	P	359.25	3
6	556.18	G	262.17	2
7		W	205.09	1

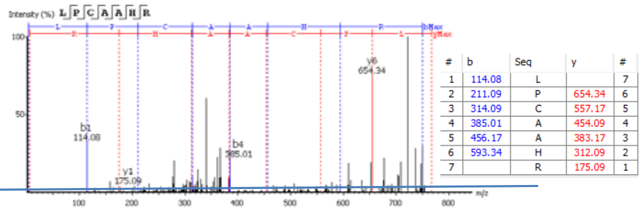

#	b	Seq	y	#
1	114.08	L		7
2	211.09	P	654.34	6
3	314.09	C	557.17	5
4	385.01	A	454.09	4
5	456.17	A	383.17	3
6	593.34	H	312.09	2
7		R	175.09	1
