# Supplementary material for: Characterisation of Endogenous Peptides Present in Virgin Olive Oil
Source: Int J Mol Sci. 2022 Feb 2;23(3):1712. doi: 10.3390/ijms23031712 (PMC8836281; doi:10.3390/ijms23031712)
Supplement: Supplementary file 1 [file ijms-23-01712-s001.zip › 1. Supplemental Material S2.pdf]

Supplemental data S2. Proteins identified as sources of endogenous peptides detected in VOO, with -10logP score above 30. Protein coverage, supporting peptides and best peptide-spectrum matches (PSM) are shown. Protein -10lgP score, sum of the -10lgP scores of the protein's supporting peptides; Coverage (%), percentage of the protein sequence that is covered by the supporting peptides; Area Sample, protein relative abundance; #Peptides, number of high-confidence supporting peptides; #Unique, number of high confidence supporting peptides that are mapped to only one protein group; PTM, posttranslational modifications. Description, as seen in the FASTA data base.

Protein List

Protein Group	Protein ID	Accession	-10lgP	Coverage (%)	Coverage (%) Sample 1	Area Sample 1	#Peptides	#Unique	#Spec Sample 1	PTM	Avg. Mass	Description
**1**	1	OE9A032471P1	155.75	9	9	2.8186E6	6	6	8	N	51572	OE9A032471P1
**1**	2	OE9A042009P1	155.75	9	9	2.8186E6	6	6	8	N	51725	OE9A042009P1
**165**	11	OE9A085162P3	93.56	4	4	1.0776E6	1	1	1	N	40777	OE9A085162P3
**165**	12	OE9A085162P1	93.56	4	4	1.0776E6	1	1	1	N	44093	OE9A085162P1
**165**	13	OE9A085162P2	93.56	3	3	1.0776E6	1	1	1	N	55757	OE9A085162P2
**3**	3	OE9A001718P2	75.15	7	7	4.5393E5	2	2	3	N	47253	OE9A001718P2
**3**	4	OE9A001718P1	75.15	7	7	4.5393E5	2	2	3	N	49955	OE9A001718P1
**9**	245	OE9A057915P1	43.19	7	7	4.0106E6	1	1	1	N	21805	OE9A057915P1
**2**	82	OE9A117792P1	43.16	2	2	6.1091E6	1	1	1	Y	38475	OE9A117792P1
**166**	177	OE9A087501P1	39.01	4	4	3.4884E7	1	1	1	N	27647	OE9A087501P1
**40**	222	OE9A013295P1	32.74	1	1	9.6294E5	1	1	2	N	72709	OE9A013295P1
**40**	223	OE9A042038P1	32.74	1	1	9.6294E5	1	1	2	N	75341	OE9A042038P1
**40**	224	OE9A062197P1	32.74	1	1	9.6294E5	1	1	2	N	75780	OE9A062197P1
**41**	244	OE9A106456P1	31.66	1	1	7.3958E4	1	1	1	N	86941	OE9A106456P1
**93**	239	OE9A058866P1	31.36	1	1	4.1833E6	1	1	1	N	108486	OE9A058866P1
**167**	249	OE9A054657P1	30.94	3	3	0	1	1	1	N	30439	OE9A054657P1
**167**	250	OE9A104108P1	30.94	2	2	0	1	1	1	N	41131	OE9A104108P1
**42**	289	OE9A026632P1	30.30	4	4	4.3063E5	1	1	1	N	30682	OE9A026632P1
**168**	293	OE9A100476P1	30.09	2	2	2.2457E5	1	1	1	N	38275	OE9A100476P1
**106**	101	OE9A087887P1	29.54	3	3	0	1	1	1	N	28540	OE9A087887P1
**total 20 proteins**												

OE9A032471P1

back to list

| Protein Coverage | Supporting Peptides | Best Unique PSM |

Protein Coverage:

```
  1 MAKICLLSLS LVLLFSLSFA QQQLKGQCQI QKINALQPQY KIQSEGGVTE SYDYNDDQFQ CAGAAFLRHT IYRRSLLLPS
 81 YTSSPLLAYA VKGSGILGIM ISGCPETFES SQESQQESHG QGQRFRDRHQ KIGNFRQGDI IAVPAGAAHW IYNSGNEELV
161 LVVLQDTSNN VNQLDDIPRR FFLAGNPRSG QEQEQQQPWQ PEKQYSRFDN VFKGFAVQVL AEAFNVDLET AKKLQGENDQ
241 RGHMVNIEQG LQVISPPFTG EEQQYGEQNR RANGLEETIC TARVRQNIDN PSRADIYNPR AGRFTTANSL TLPILSFLQL
321 SAARGVLHRK AIMSPHWYMN CHTIVYATKG NARMQIVDNR GQAVFDDTIQ EGQVVVVPHN FAVVKQAGDQ GFEWVEFNTN
401 ENAMINTISG RTSSFRGLPV DVIVNAYQVS RKVAEQLKFN RQETLIFSGG ESSGQPRVSS V
```

Supporting Peptides:

Peptide	Uniq	-10lgP	Mass	Length	ppm	m/z	z	RT	Fraction	Scan	Source File	Area Sample 1	#Feature	#Feature Sample 1	Start	End	PTM	AScore	Fou By
L.VVLQDTSNNVNQLD.D	Y	77.73	1557.7634	14	5.9	779.8878	2	24.96	1	4137	P2860-Aislado informe 2.raw	3.4433E5	1	1	162	175			PEA DE
L.VVLQDTSNNVNQLDDIPRRFFLA.G	Y	63.26	2673.3875	23	5.2	892.1344	3	48.98	1	10542	P2860-Aislado informe 2.raw	0	0	0	162	184			PEA DE
L.IFSGGESSGQPR.V	Y	57.53	1220.5785	12	6.8	611.2961	2	22.47	1	3436	P2860-Aislado informe 2.raw	1.2435E5	1	1	446	457			PEA DE

total 6 peptides

Peptide	Uniq	-10lgP	Mass	Length	ppm	m/z	z	RT	Fraction	Scan	Source File	Area Sample 1	#Feature	#Feature Sample 1	Start	End	PTM	AScore	Fou By
Q.DTSNNVNQLDDIPRR.F	Y	46.89	1755.8500	15	5.1	586.2892	3	26.25	1	4526	P2860-Aislado informe 2.raw	1.5401E5	1	1	166	180			PEAI DE
L.QDTSNNVNQLDDIPRR.F	Y	33.36	1883.9086	16	8.0	628.9771	3	26.36	1	4524	P2860-Aislado informe 2.raw	2.0584E6	1	1	165	180			PEAI DE
M.INTISGR.T	Y	31.90	759.4239	7	8.6	380.7197	2	20.53	1	2900	P2860-Aislado informe 2.raw	1.3753E5	1	1	405	411			PEAI DE
**total 6 peptides**																			

Best Unique PSM (Scan 4137, m/z=779.8878, z=2, RT=24.96, ppm=5.9):

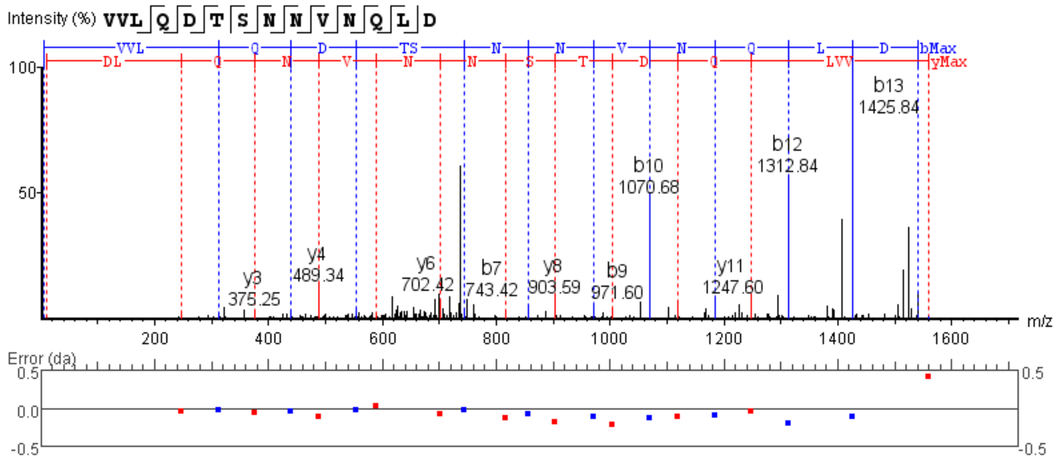

OE9A042009P1

back to list

| Protein Coverage | Supporting Peptides | Best Unique PSM |

Protein Coverage:

```
  1 MAKIICLLSL SLVLLFSLSF AQQQLKGQCQ IQKINALQPQ YKIQSEGGVT ESYDYNDDQF RCAGAAFLRH TIYRRSLLLP
 81 SYTSSPLLAY AVKGSGILGI MISGCPETFE SSQESQQESQ GQGQRFRDRH QKIGNFLQGD IIAFPAGAAH WIYNSGNEEL
161 VLVVLQDTSN NVNQLDDIPR RFFLAGNPRS GQEQEQQQLW QPEKQYSRFD NVFKGFAVQV LAEAFNVDLE TAKKLQGEND
241 QRGHMVNIEQ GLQVISPPFT GEEQQYGEQN RRANGLEETI CTARVRQNID NPSRADIYNP RAGRFTTANS LTLPILSFLQ
321 LSAARGVLHR KAIMSPHWYM NCHTIVYATK GNARMQIVDN RGQAVFDDTI QEGQVVVVPH NFAVVKQAGD QGFEWVEFNT
401 NENAMINTIS GRTSSFRGLP VDVIVNAYQV SRKVAEQLKF NRQETLIFSG GESSGQPRVS SV
```

Supporting Peptides:

Peptide	Uniq	-10lgP	Mass	Length	ppm	m/z	z	RT	Fraction	Scan	Source File	Area Sample 1	#Feature	#Feature Sample 1	Start	End	PTM	AScore	Fou By
L.VVLQDTSNNVNQLD.D	Y	77.73	1557.7634	14	5.9	779.8878	2	24.96	1	4137	P2860-Aislado informe 2.raw	3.4433E5	1	1	163	176			PEAI DE
L.VVLQDTSNNVNQLDDIPRRFFLA.G	Y	63.26	2673.3875	23	5.2	892.1344	3	48.98	1	10542	P2860-Aislado informe 2.raw	0	0	0	163	185			PEAI DE
L.IFSGGESSGQPR.V	Y	57.53	1220.5785	12	6.8	611.2961	2	22.47	1	3436	P2860-Aislado informe 2.raw	1.2435E5	1	1	447	458			PEAI DE
Q.DTSNNVNQLDDIPRR.F	Y	46.89	1755.8500	15	5.1	586.2892	3	26.25	1	4526	P2860-Aislado informe 2.raw	1.5401E5	1	1	167	181			PEAI DE
L.QDTSNNVNQLDDIPRR.F	Y	33.36	1883.9086	16	8.0	628.9771	3	26.36	1	4524	P2860-Aislado informe 2.raw	2.0584E6	1	1	166	181			PEAI DE
M.INTISGR.T	Y	31.90	759.4239	7	8.6	380.7197	2	20.53	1	2900	P2860-Aislado informe 2.raw	1.3753E5	1	1	406	412			PEAI DE
**total 6 peptides**																			

Best Unique PSM (Scan 4137, m/z=779.8878, z=2, RT=24.96, ppm=5.9):

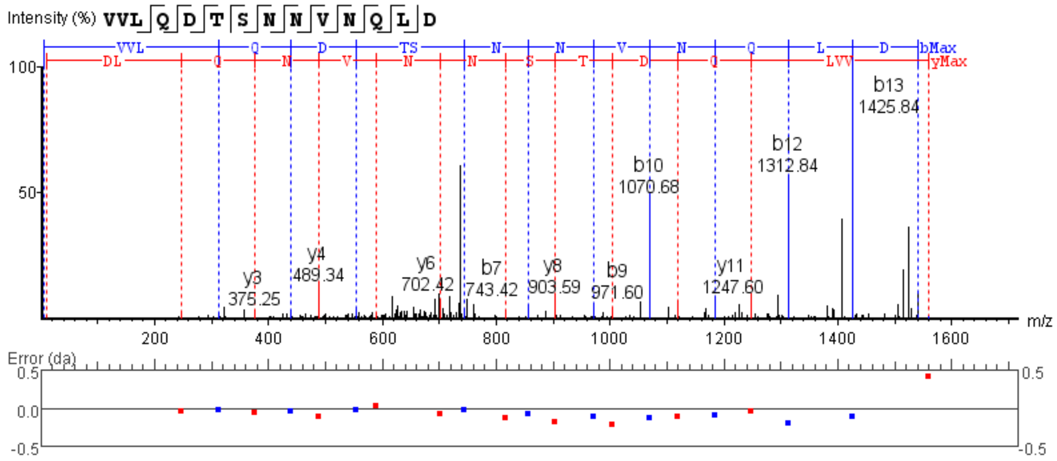

OE9A085162P3

back to list

| Protein Coverage | Supporting Peptides | Best Unique PSM |

Protein Coverage:

```
  1 MRQQEQGSES QKQFFDRHQK VRQFRQGDVL ALPAGITLWL YNNGQEPLVT VSLLDTANEM NQLDLQFRNF FLAGNRNPQQ
 81 SQGQQQEESQ SEGESPINNI FYGFPDKVLA DVYNVEPETI RKLKGEQDQR GRIVKAERFN VVLPREGQEE EQEGQQGQGG
161 RNGLEETLCT LRLRENLGRP SRADVYNPRA GRLATLNSQT LPILNYLQLS AVKGVLYRNA IMAPHWNVNA HSVIYITRGS
241 SRLQVVGHSG NLVFDGEVKE NQLIIIPQSF VVIKKAGDQG CEWIAFKTND NAMISPLAGR LSAFRSMPVD VLANAYRVSK
321 QEAQVLKFSR DESTLFSSSS SSMSLEKPKA MEYARDVIET VI
```

Supporting Peptides:

Peptide	Uniq	-10lgP	Mass	Length	ppm	m/z	z	RT	Fraction	Scan	Source File	Area Sample 1	#Feature	#Feature Sample 1	Start	End	PTM	AScore	Found By
L.LDTANEMNQLDLQFR.N	Y	93.56	1806.8571	15	8.3	904.4365	2	34.18	1	6794	P2860-Aislado informe 2.raw	1.0776E6	1	1	54	68			PEAKS DB
**total 1 peptides**																			

Best Unique PSM (Scan 6794, m/z=904.4365, z=2, RT=34.18, ppm=8.3):

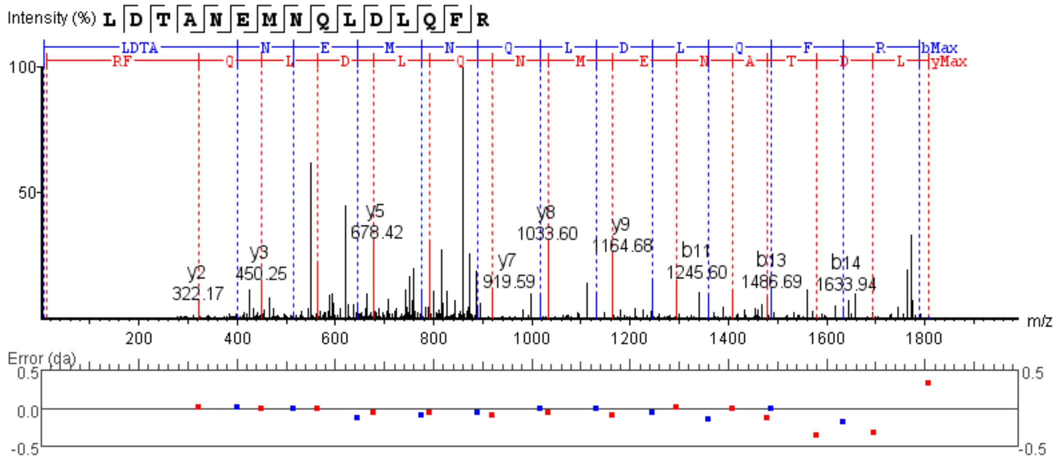

OE9A085162P1

back to list

| Protein Coverage | Supporting Peptides | Best Unique PSM |

Protein Coverage:

```
  1 MRQQEQGSES QKQFFDRHQK VRQFRQGDVL ALPAGITLWL YNNGQEPLVT VSLLDTANEM NQLDLQFRVR ISEISWLVSN
 81 IIRDFIEIFT IFFFQNFFLA GNRNPQQSQG QQQEESQSEG ESPINNIFYG FPDKVLADVY NVEPETIRKL KGEQDQRGRI
161 VKAERFNVVL PREGQEEEQE GQQGQGGRNG LEETLCTLRL RENLGRPSRA DVYNPRAGRL ATLNSQTLPI LNYLQLSAVK
241 GVLYRNAIMA PHWNVNAHSV IYITRGSSRL QVVGHSGNLV FDGEVKENQL IIIPQSFVVI KKAGDQGCEW IAFKTNDNAM
321 ISPLAGRLSA FRSMPVDVLA NAYRVSKQEA QVLKFSRDES TLFSSSSSSM SLEKPKAMEY ARDVIETVI
```

Supporting Peptides:

Peptide	Uniq	-10lgP	Mass	Length	ppm	m/z	z	RT	Fraction	Scan	Source File	Area Sample 1	#Feature	#Feature Sample 1	Start	End	PTM	AScore	Found By
L.LDTANEMNQLDLQFR.V	Y	93.56	1806.8571	15	8.3	904.4365	2	34.18	1	6794	P2860-Aislado informe 2.raw	1.0776E6	1	1	54	68			PEAKS DB
**total 1 peptides**																			

Best Unique PSM (Scan 6794, m/z=904.4365, z=2, RT=34.18, ppm=8.3):

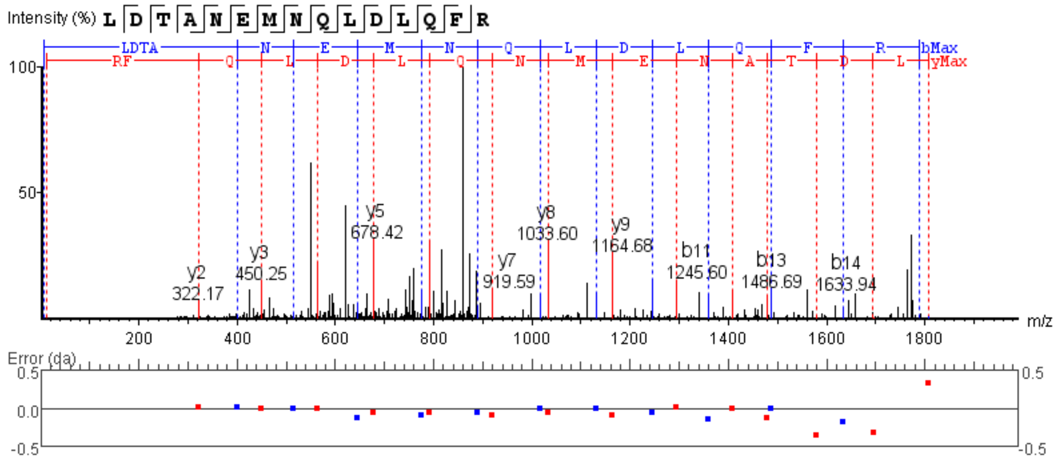

OE9A085162P2

[back to list]

| Protein Coverage | Supporting Peptides | Best Unique PSM |

Protein Coverage:

```
  1 MAKLSLHFLS FTLLFLFGGC LAQLGSQQQF LWQKLQQQQQ HRLRAKTDCR IERLTAQEPT RRYDSEAGYA EYWDPNNKQF
 81 ECAGVAAVRT SIQKNGLFLP HYNNVAQLIY VVQGKGLLGA VIPGCAETFE TEMRQQEGQS ESQKQFFDRH QKVRQFRQGD
161 VLALPAGITL WLYNNGQEPL VTVSLLDTAN EMNQLDLQFR NFFLAGNRNP QQSQGQQQEE SQSEGESPIN NIFYGFPDKV
241 LADVYNVEPE TIRKLKGEQD QRGRIVKAER FNVVLPREGQ EEEQEGQQGQ GGRNGLEETL CTLRLRENLG RPSRADVYNP
321 RAGRLATLNS QTLPILNYLQ LSAVKGVLYR NAIMAPHWNV NAHSVIYITR GSSRLQVVGH SGNLVFDGEV KENQLIIIPQ
401 SFVVIKKAGD QGCEWIAFKT NDNAMISPLA GRLSAFRSMP VDVLANAYRV SKQEAQVLKF SRDESTLFSS SSSSMSLEKP
481 KAMEYARDVI ETVI
```

Supporting Peptides:

Peptide	Uniq	-10lgP	Mass	Length	ppm	m/z	z	RT	Fraction	Scan	Source File	Area Sample 1	#Feature	#Feature Sample 1	Start	End	PTM	AScore	Found By
L.LDTANEMNQLDLQFR.N	Y	93.56	1806.8571	15	8.3	904.4365	2	34.18	1	6794	P2860-Aislado informe 2.raw	1.0776E6	1	1	186	200			PEAKS DB
**total 1 peptides**																			

Best Unique PSM (Scan 6794, m/z=904.4365, z=2, RT=34.18, ppm=8.3):

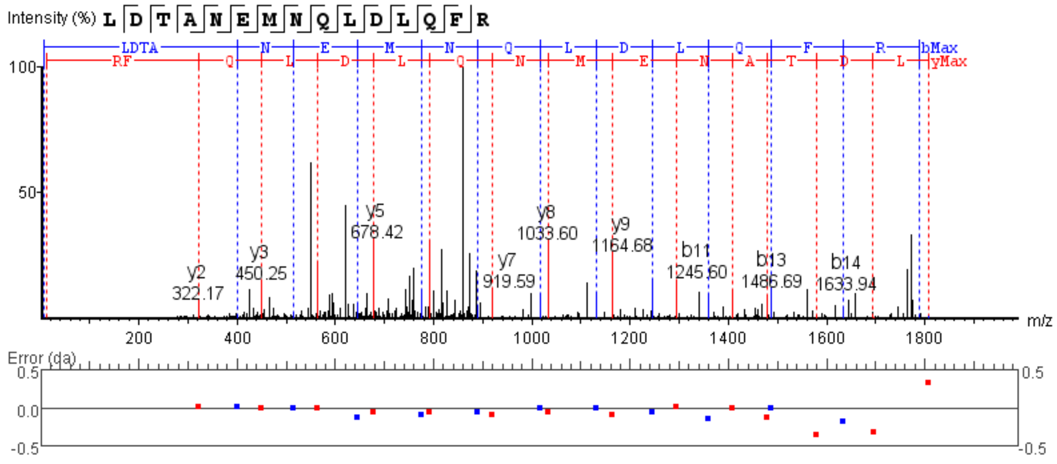

OE9A001718P2

[back to list]

| Protein Coverage | Supporting Peptides | Best Unique PSM |

Protein Coverage:

```
  1 MKQAAGHGLL FMVLFFLLPI GSLSSDHHRH KSSFRRVLIG HDSDKYAVIF DAGSTGSRVH VFRFDQNQDL LPIGNELELY
 81 VSTKPGLSSY AADPQAAASS LRPLLEEAEA VVPIWLQPDT PARLGATAGL RLVEGDAAEK ILDAVRNLFK NESHLKYKHD
161 WVSILDGVQE GTYMWVTINY LLNTLGRSYA ETVATIDLGG ASVQMAYAIS DENAAKAPHV AANETYLLQK NLKGTNYNLY
241 VHSYLKYGSK AARAEIFKAS RNSTNPCILE GYEGTYTYGG VVYRVSAPRK GANMKRCQIL TRKALKINAP CKYDSCTFNG
321 IWNGGGGDGE KNLYLSSSFY WTALDSGILK QNATGGRILV NAYKDTAKTA CSTKFMDVKS KFPDVQEENI PYLCMDLVYI
401 YTLLVDGLGE LKFLLILPEE IINYTNTA
```

Supporting Peptides:

Peptide	Uniq	-10lgP	Mass	Length	ppm	m/z	z	RT	Fraction	Scan	Source File	Area Sample 1	#Feature	#Feature Sample 1	Start	End	PTM	AScore	Found By
E.AVVPIWLQPDTPAR.L	Y	57.79	1561.8616	14	9.6	781.9397	2	38.09	1	7972	P2860-Aislado informe 2.raw	4.3861E5	1	1	110	123			PEAKS DB
R.VHVFRFDQNQDLLPIGN.E	Y	34.71	2011.0275	17	8.8	671.3506	3	48.03	1	10402	P2860-Aislado informe 2.raw	1.5322E4	1	1	59	75			PEAKS DB
**total 2 peptides**																			

Best Unique PSM (Scan 7972, m/z=781.9397, z=2, RT=38.09, ppm=9.6):

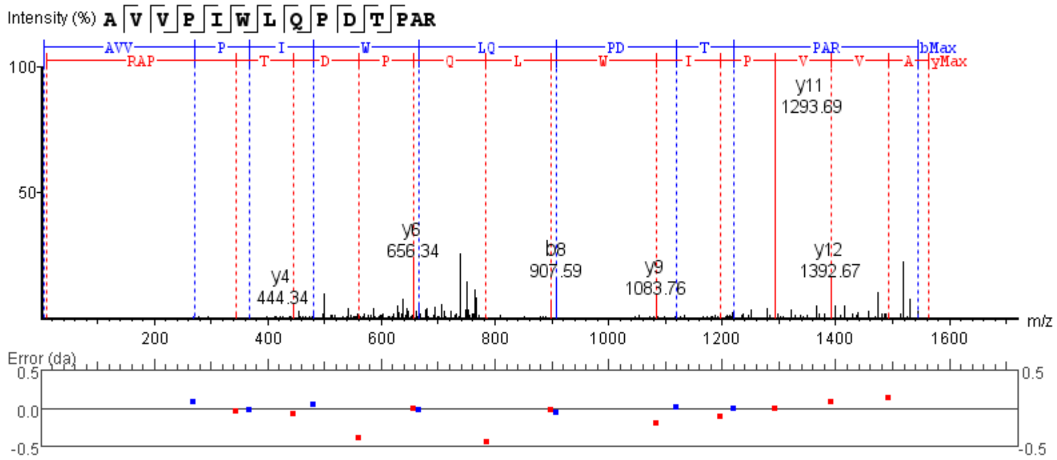

OE9A001718P1

[back to list]

| **Protein Coverage** | **Supporting Peptides** | **Best Unique PSM** |

Protein Coverage:

```
  1 MKQAAGHGLL FMVLFFLLPI GSLSSDHHRH KSSFRRVLIG HDSDKYAVIF DAGSTGSRVH VFRFDQNQDL LPIGNELELY
 81 VSTKPGLSSY AADPQAAASS LRPLLEEAEA VVPIWLQPDT PARLGATAGL RLVEGDAAEK ILDAVRNLFK NESHLKYKHD
161 WVSILDGVQE GTYMWVTINY LLNTLGRSYA ETVATIDLGG ASVQMAYAIS DENAAKAPHV AANETYLLQK NLKGTNYNLY
241 VHSYLKYGSK AARAEIFKAS RNSTNPCILE GYEGTYTYGG VVYRVSAPRK GANMKRCQIL TRKALKINAP CKYDSCTFNG
321 IWNGGGGDGE KNLYLSSSFY WTALDSGILK QNATGGRILV NAYKDTAKTA CSTKFMDVKS KFPDVQEENI PYLCMDLVYI
401 YTLLVDGLDL NASQKVEVVK DVKYKNSEVE ASWPLGCAID VTSSLTSKGL IEKI
```

Supporting Peptides:

Peptide	Uniq	-10lgP	Mass	Length	ppm	m/z	z	RT	Fraction	Scan	Source File	Area Sample 1	#Feature	#Feature Sample 1	Start	End	PTM	AScore	Found By
E.AVVPIWLQPDTPAR.L	Y	57.79	1561.8616	14	9.6	781.9397	2	38.09	1	7972	P2860-Aislado informe 2.raw	4.3861E5	1	1	110	123			PEAKS DB
R.VHVFRFDQNQDLLPIGN.E	Y	34.71	2011.0275	17	8.8	671.3506	3	48.03	1	10402	P2860-Aislado informe 2.raw	1.5322E4	1	1	59	75			PEAKS DB
**total 2 peptides**																			

Best Unique PSM (Scan 7972, m/z=781.9397, z=2, RT=38.09, ppm=9.6):

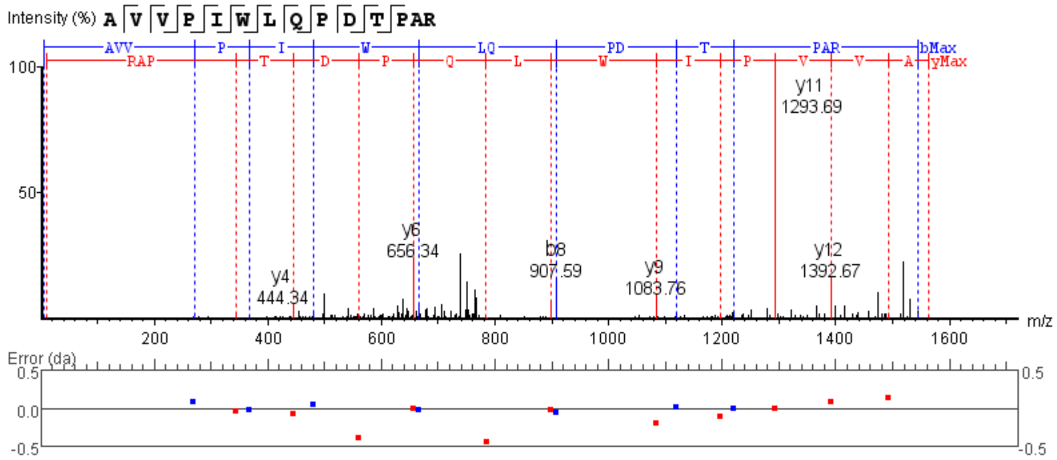

OE9A057915P1

[back to list]

| **Protein Coverage** | **Supporting Peptides** | **Best Unique PSM** |

Protein Coverage:

1 ERVPVVKDET VVQDNDFEKR TSASVASTLQ SPPNKAEGTD NSNANAQNGT CSARSLLKSA SISASKCIGV KGTNNTEEES
81 IAEHSEETQI YHSVVPYIDF ESISGRSFRK LDYPKNSVMP PSISPFTLVL GCHAGGKAGG KGGEGGKGGV GGVGAG**KGGG**
161 **GGSGSAGGGG S**AKGSDGIGA SSGMMKAPGG GGATISRNLK AILKDILLKP GLPAGEKAGK

Supporting Peptides:

Peptide	Uniq	-10lgP	Mass	Length	ppm	m/z	z	RT	Fraction	Scan	Source File	Area Sample 1	#Feature	#Feature Sample 1	Start	End	PTM	AScore	Found By
G.KGGGGGSGSAGGGGS.A	Y	31.14	1048.4534	15	-4.9	525.2275	2	35.95	1	7525	P2860-Aislado informe 2.raw	4.0106E6	1	1	157	171			PEAKS DB
**total 1 peptides**																			

Best Unique PSM (Scan 7525, m/z=525.2275, z=2, RT=35.95, ppm=-4.9):

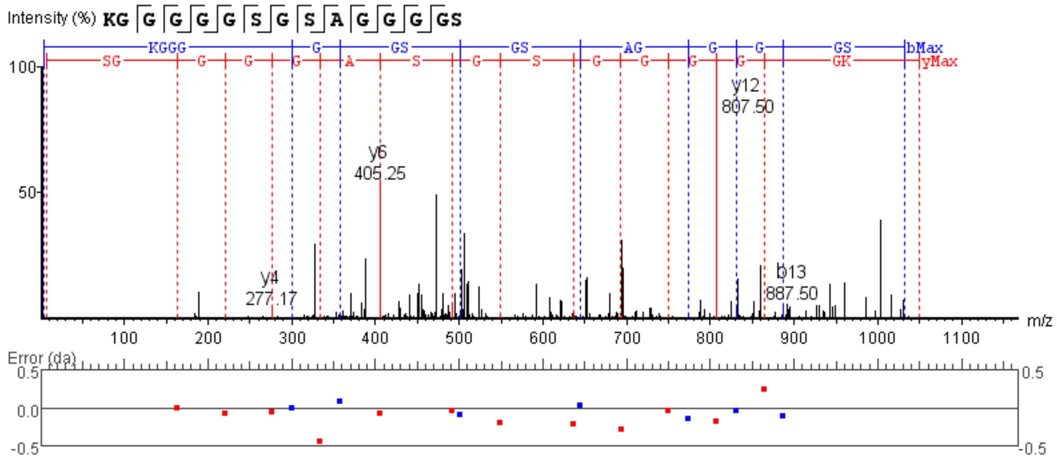

OE9A117792P1

[back to list]

| **Protein Coverage** | **Supporting Peptides** | **Best Unique PSM** |

Protein Coverage:

1 MVSPSPASAV AVAWRQVRHE AIQVEVADWS VRALRGKESK ELVVDGGSAL CESWAAACRR VRDESRIRQW LHLLQSSLRV
81 QLGFVFLFWV RQYQLLSPKR SFFFIINAFA AGVILLIGFI HVLPDAFESL TSPHLSSVAM VSAIGTLMVD AYATSHYNKK
161 SSSMAQIASG DEGGVVPVHT HPTHGHAHGS ASVETDSGET QVLRHRVISQ VLEIIVHSVI IGIALGASES PKTIKPLIAA
241 LTFHQFFEGI GLGGCITQAK FKSRAVVIMA IFFSLTTPIG IVIGIGISNI YSETSPTALI IEGIFNSASA GILIYMALVD
321 LLSADFISPK MQGNGKLQSG ANVS**LLLGAG CM**SLLAKWA

Supporting Peptides:

Peptide	Uniq	-10lgP	Mass	Length	ppm	m/z	z	RT	Fraction	Scan	Source File	Area Sample 1	#Feature	#Feature Sample 1	Start	End	PTM	AScore	Found By
S.LLLGAGCM(+15.99).S	Y	34.51	792.3874	8	1.4	397.1985	2	33.76	1	7091	P2860-Aislado informe 2.raw	6.1091E6	1	1	345	352	Oxidation (M)	M8:Oxidation (M):1000.00	PEAKS DB
**total 1 peptides**																			

Best Unique PSM (Scan 7091, m/z=397.1985, z=2, RT=33.76, ppm=1.4):

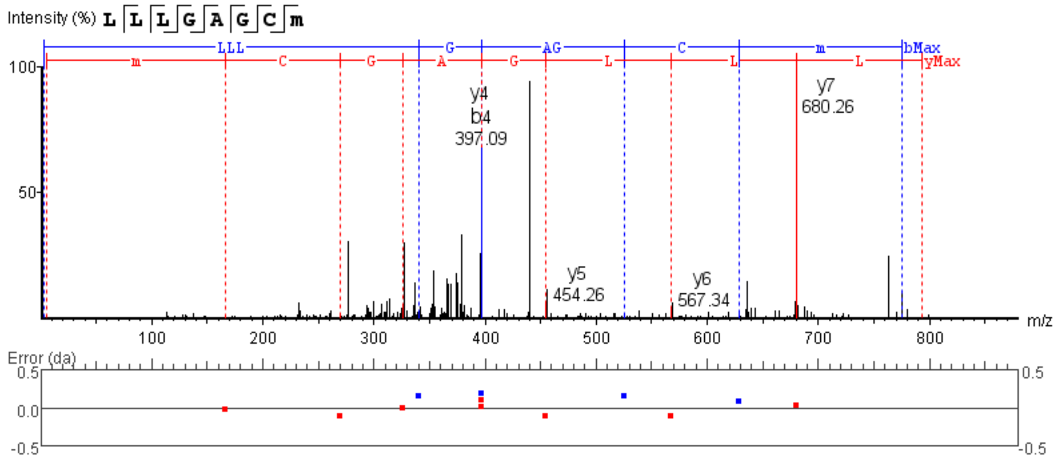

OE9A087501P1

[back to list]

| **Protein Coverage** | **Supporting Peptides** | **Best Unique PSM** |

Protein Coverage:

1 MPSCFPITGK TTLAQNVIFD PSPRSMENPN RVLIVMNGLK DVSIELLEWV LKNFTFHDCG TITIFGVSPW LNIPLSAKTW
81 SDIWSMDLED LSIVKERIEW KNDPKYQKVL RLVDLCQKYG VVPEIRTEMG HPLRLLVVEQ ISSLNATLVV FDKYHDRKNI
161 EYYAEKVPCN MVVVNDNGEV ELIKKRSCMD SDVENTPTTI AESSDAAAMA **NCSTSIISG**Q LKKRLKSKSR GKRDKEMKYP
241 DTD

Supporting Peptides:

Peptide	Uniq	-10lgP	Mass	Length	ppm	m/z	z	RT	Fraction	Scan	Source File	Area Sample 1	#Feature	#Feature Sample 1	Start	End	PTM	AScore	Found By
A.NCSTSIISG.Q	Y	39.01	880.3961	9	9.5	441.2062	2	35.88	1	7180	P2860-Aislado informe 2.raw	3.4884E7	1	1	211	219			PEAKS DB
total 1 peptides																			

Best Unique PSM (Scan 7180, m/z=441.2062, z=2, RT=35.88, ppm=9.5):

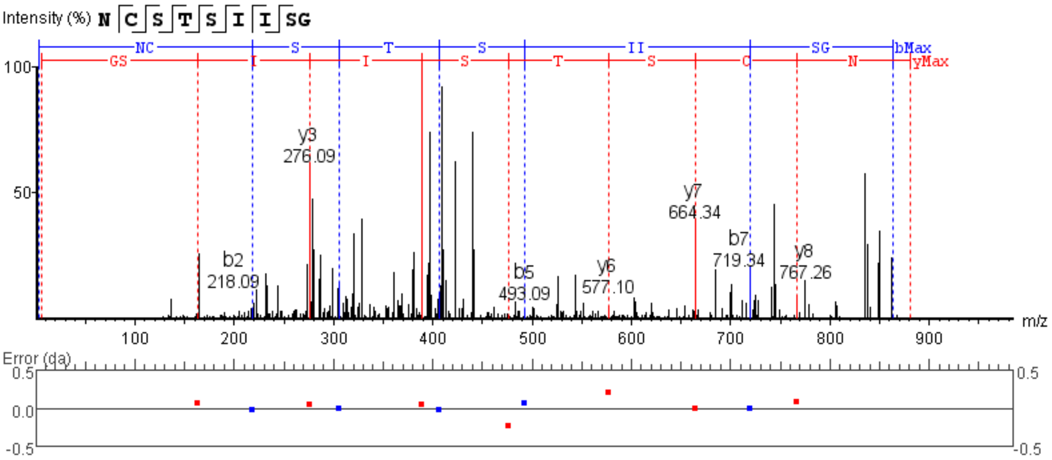

OE9A013295P1

back to list

| Protein Coverage | Supporting Peptides | Best Unique PSM |

Protein Coverage:

1 MHPRKLLVLL IFISLSFHPD YVLPENVNLD FPFFTLRNIT LLGDSYIRNG VVGLTRELEV PSSSSGSVIY NYPVAFFDPE
81 TNITASFSTR FSFSIDNVNP SSFGDGLTFF LSPNNQTLGS PGGYLGLVNS SQLTKERFIA IEFDTRQDLL FNDPDENHVG
161 LDINSLNSIK TANSMIQGVN LKGGNLITAW IDYENEKKKL EIFLSYSSFK PEEPLLRVGV DLSDYLKEFM YVG**FSASTEG**
241 **S**TELHFIQNW NFRTLGFRPI RPKIHPYNVS ESSVPLRPPI TVSDSGNRHH KRIGLGLGIG FPAFFCTVLV GFGWISIKKW
321 KGIKSERTIK AEMVTGPRQF SYKELKSATR GFHSSRILGH GAFGTVFKAF FMDLGTISAV KRSKHTHEGK TEFLSELSII
401 ASLRHKNLVQ LQGWCVEKGE LLLVYDFMPS GSLDKVLYQE SENGNPLKWT YRYNSAVGLA SVLTYLHQEC EQQVIHRDIK
481 TSNVMLDGNY NARLGDFGLA RLMDHGTSPV STLTAGTMGY LAPEYLQYGA ATEKTDVFSY GVVILELACG RRPIEREDES
561 QKPVNLVDWV WRKYSEGKLL EAADQRMNGE FKEEEMKRLL LIGLSCANPN SSERPSMRRV FQILNNEADP VVVPKVKPSL
641 ISRTACH

Supporting Peptides:

Peptide	Uniq	-10lgP	Mass	Length	ppm	m/z	z	RT	Fraction	Scan	Source File	Area Sample 1	#Feature	#Feature Sample 1	Start	End	PTM	AScore	Found By
G.FSASTEGS.T	Y	32.74	784.3239	8	0.5	393.1665	2	25.27	1	4197	P2860-Aislado informe 2.raw	9.6294E5	2	2	234	241			PEAKS DB
total 1 peptides																			

Best Unique PSM (Scan 4197, m/z=393.1665, z=2, RT=25.27, ppm=0.5):

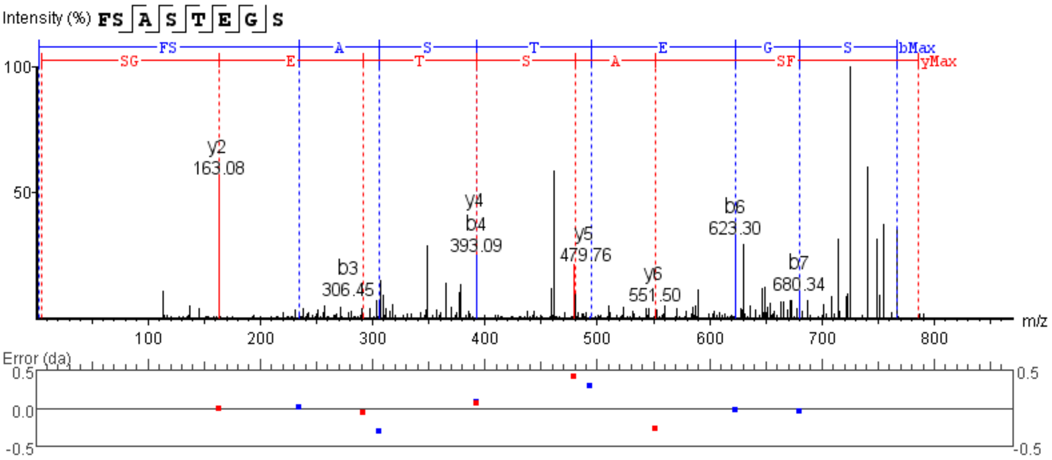

OE9A042038P1

back to list

| **Protein Coverage** | **Supporting Peptides** | **Best Unique PSM** |

Protein Coverage:

```
  1  MSPRKLLVFL ILINLSVHPD YVSSANVNLD FPFFTFRNIT LLGDSYLRNG VIGLTRELGV PSSSSGTVIY NHPVAFFDPE
 81  TNITASFSTR FSFSIDNVNP SSFGDGLAFF LSPNNQTLGS PGGYLGLVNS SEFSKNRFIA IEFDTKQDLH FNDPDENHVG
161  LDINSLISIK TANSMIRGVN LKSGNLITAW IDYENEKKKF EIFLSYSSFK PEEPLLRVSI DLSDYLKEFM YVGFSASTEG
241  STELHCIENW SFRTMGFRPI RPKVQPHSVS ESSVPLKPPI TVSGSGNRHH KRIGLGLGIG FPAFFGAVLV IFGWISVKKW
321  KGTKSERIIK AELVTGPRQF SYKELKSATR GFHSSRILGH GAFGTVYKAF FMDLGTISAV KRSKHTHEGK TEFLSELSII
401  ASLRHKNLVQ LQGWCVEKGE LLLVYDFMPN GSLDKVLYQE SENNNPLKWT YRYYIAVGLA SVLTYLHQEC EQQVIHRDIK
481  TSNVMLDGNY NARLGDFGLA RLMDHGTSPV STLTAGTMGY LVPEYLQYGT ATEKTDVYSY GVVVLELACG RRPIEREDES
561  QKQVNLVDWV WRKYSEGKLI EAADQRMNGE FKEEEMRRLL LIGLSCANPD SSERPSMRRV LQILNNEADP VIVPKVKPSL
641  IFSNSLPLSI DEIVSDCEEC SSPGSELEIK VY
```

Supporting Peptides:

Peptide	Uniq	-10lgP	Mass	Length	ppm	m/z	z	RT	Fraction	Scan	Source File	Area Sample 1	#Feature	#Feature Sample 1	Start	End	PTM	AScore	Found By
G.FSASTEGS.T	Y	32.74	784.3239	8	0.5	393.1665	2	25.27	1	4197	P2860-Aislado informe 2.raw	9.6294E5	2	2	234	241			PEAKS DB
total 1 peptides																			

Best Unique PSM (Scan 4197, m/z=393.1665, z=2, RT=25.27, ppm=0.5):

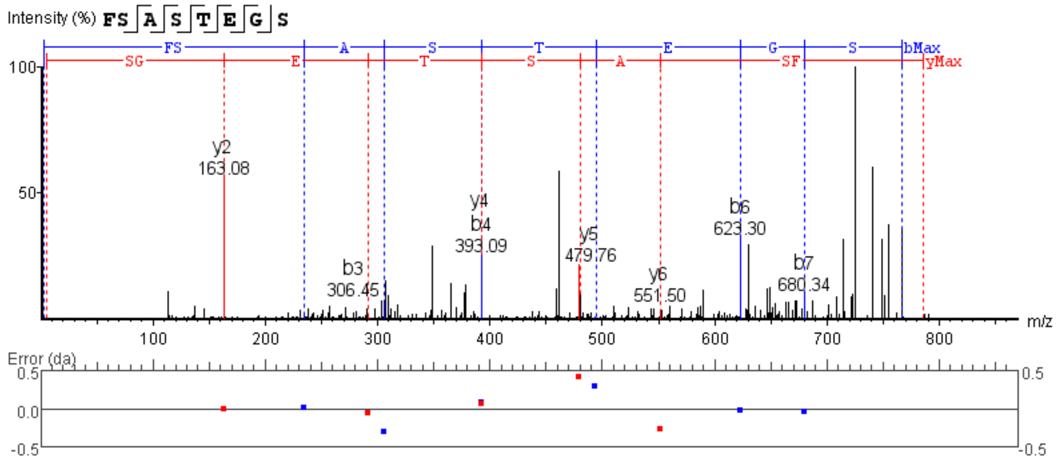

OE9A062197P1

back to list

| **Protein Coverage** | **Supporting Peptides** | **Best Unique PSM** |

Protein Coverage:

```
  1  MNSRKLLVVS TLLFLFNFWQ HPLLVSSENV NLDFPVFSFR NFTILGDSYL RNGVFGLTRE LEVPTSSSGC VIYNHPIEFF
 81  DRETNITASF STRFSFSIGN VNPSSFGDGL TFFISPNNQT VGNAGGYLGL VNSSQLTKNR FTAIEFDTRQ DLHFNDPDDN
161  HVGLDINSLI SIKTANPTLQ SVNLKSGNLI TAWIDYMNEK KKLDVFLSYS SFKPEKPLLN VNVDLSGYLN EFMYVGFSAS
241  TEGSTELHYI ENWSFQTMGF HPVRPRIHPH NVSDSSVPHT PPTPVSDSGN KNHKRIRLGL GVGFPAFFCS VLVVFGWISF
321  KKWRESRTEK CLKAELVTGP RQYSYKEIKS ATRGFHSSRI VGHGAFGTVY KAFFTDLGTI SAVKRSKHAH EGKTEFLAEL
401  SIIACLRHKN LVPLQGWCVE KGELLLVYEF MPHGSLDSLL YSDSEHGNPL QWSYRYNIAV GLASVLTYLH QECEQQVIHR
481  DVKTSNIMLD ANYNARLGDF GLARIMDHGK SPVSTLTAGT MGYLAPEYLQ YGKATEKTDV FSYGVVILEL ASGRRPIERE
561  TGSQKMVNLV DWAWMLHSEG KIIEAADKNM NGEYDEKEMK KLLLIGLSCA NPDSMERPSM RAVLQILNNE AEPMVVPKAK
641  PTLTFSNSLP LSIDDIVLDC EDIVIPESQF EIRPE
```

Supporting Peptides:

Peptide	Uniq	-10lgP	Mass	Length	ppm	m/z	z	RT	Fraction	Scan	Source File	Area Sample 1	#Feature	#Feature Sample 1	Start	End	PTM	AScore	Found By
G.FSASTEGS.T	Y	32.74	784.3239	8	0.5	393.1665	2	25.27	1	4197	P2860-Aislado informe 2.raw	9.6294E5	2	2	237	244			PEAKS DB
total 1 peptides																			

Best Unique PSM (Scan 4197, m/z=393.1665, z=2, RT=25.27, ppm=0.5):

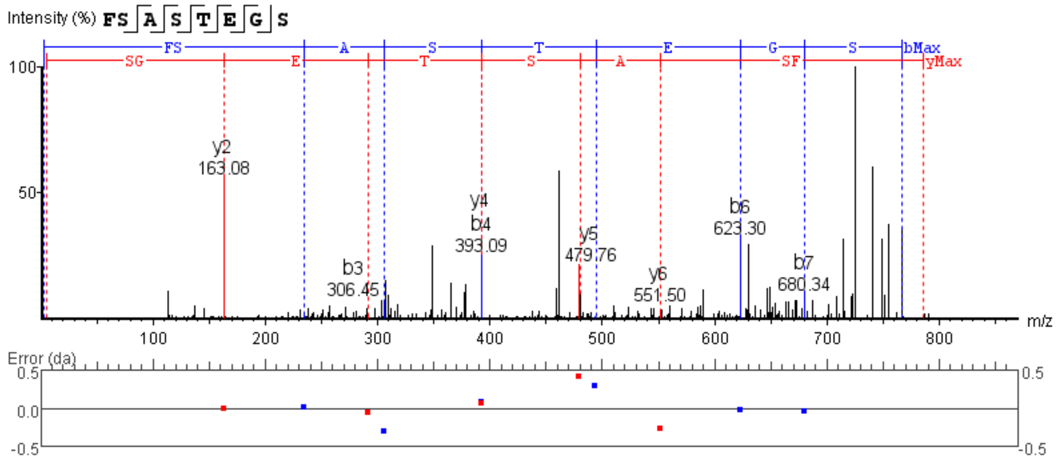

OE9A106456P1

back to list

| Protein Coverage | Supporting Peptides | Best Unique PSM |

Protein Coverage:

```
  1 MEIEDDSGGQ DLKSGTALPS RSGKSTMVGL DKELIEILNW LDGNSSARDT LSIVGMPGIG KTTFAGKIYD HQRVQEIFHV
 81 RAWVTVSQNY DERKILLDLL ESMKKLSGEM HNLETSDLKD KLHKNLKGRR YLIVIDDIWD TKVWNDVNRL FPVDQKRSRI
161 ILTTRLESVA VHVNSSRFAF RRMRFLNGVE SWNLFCKVFG GDCCPGELYQ TGTEIAKKCG GLPLAIVLIG GLLSKSNQTQ
241 SYWDHVAKNL SSVIASHDDQ SSKILHLSYN NLPHHLRECF LYMGIFPEDY EILASKLVKL WTAEGFIETE TSKSLEDVAK
321 EYLKNLVERS LILVSKRSSD GEIKTCKIHD LLREFCVREA KESFFHVTDT SLRDLPTNIS TSHITDPSLR SLPDKIISLR
401 RVSIHPNTQT SHPHADRLPT STVRSVLNFD PTLSLSVEFL NSSLIRVLDT VNGNSSDFPT EIVKLLNLRY LSCLNGVREL
481 PSPIHELRNL QTLILSCMES SFRLPLEIWR MTQLRHVHLD LVSLPDPPIE RVEEENPLVV LHDLQSLSTV MNFKFVENIL
561 RRIPNLKKLG ILFDEIGEDW SDYCLNNLAL LQNLEALKCL FNWPYKPFLQ KIIFPPSLKK LTLRGCFLPW EDMTIIGSLE
641 KLQVLKLKRK AFSGTVWEPK EGEFLELEFL LLEENNLEDW NAERTHFPKL EHLILDNCQR LKEVPCGIGE IPTLQKIELY
721 YCSDSLVTSA KDIYEEQQES GNEDFQVRIV EMTAEAYET
```

Supporting Peptides:

Peptide	Uniq	-10lgP	Mass	Length	ppm	m/z	z	RT	Fraction	Scan	Source File	Area Sample 1	#Feature	#Feature Sample 1	Start	End	PTM	AScore	Found By
W.LDGNSSAR.D	Y	31.66	818.3882	8	-4.9	410.1964	2	18.63	1	2491	P2860-Aislado informe 2.raw	7.3958E4	1	1	41	48			PEAKS DB
total 1 peptides																			

Best Unique PSM (Scan 2491, m/z=410.1964, z=2, RT=18.63, ppm=-4.9):

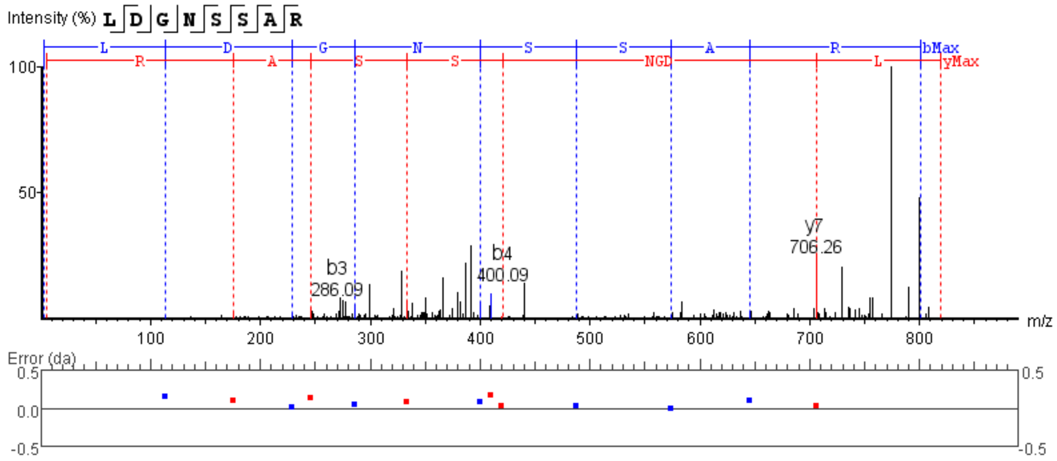

OE9A058866P1

back to list

| Protein Coverage | Supporting Peptides | Best Unique PSM |

Protein Coverage:

1 MASLASPVNL GSVSMISSCQ FEGSVCLVRR VAFRGNGNNF RRFLGGKRWR YVGICKYSVT TNYITEQGTS TSTDSTCRGS
81 KDNDTDTFLK AAPKPVLKPG SKVEPVLDMP WDEVKLKEDS SNEKTSESEE ERSKVIESLG EVLEMAEKLE KSKEPNVSVN
161 KPSGNKNAGL KNGKPVNSMA DSNSKSKTSK SVWRKGNPVA AAPKVVKEPP KQDSRIDNRG KDASHAVTPP RPPQPPQNVR
241 PQLQAKPSVA PPVSIKKPVI LKDVNAATKS SVADETNSTT AKKERKPILI DKFASKKPVV DPLIAQAVLA PPKPGKSPPP
321 GKFKDEFRKK GGPSGGSRRR MFDDDIPDED ASELDVSIPG AATARKGRKW TKASRKAARL QAAKDAAPVK VEIMEVDEDG
401 MLTEELAYNL AISEGEILGY LYSKGIKPDG VQKLSKDMVK MICREYEVEI IDTAPVRVEE MAKKKEMFDN DDLDKLEDRP
481 PVLTIMGHVD HGKTTLLDYI RKSKVAASEA GGITQGIGAY KVQVPIDGKP QICVFLDTPG HEAFGAMRAR GARVTDIAVI
561 VVAADDGIQP QTNEAIAHAK SAVVPIIVAI NKIDKDGANP DRVMQDLSSI GLMPEEWGGD IPVVKISALK GENVDDLLET
641 IMLVAELQDL KANPHRNAKG TVIEAGLDKS KGPVATFIVQ NGTLNRGDVV **VCGEAFGKA**R ALFDDKGKRI DEAGPSMPVQ
721 VIGLNNVPLA GDEFEVVGSL DVAHEKAELQ ANYLRNERIT AKAGDGKVTL SSLASAVSAG KLTGLDLHQL NIILKVDLQG
801 SIEAIRQALQ VLPQTNVTLK FLLQATGDVS TSDVDLAVAS KAIIFGFNVK APGSIKSYAD NKGVEIRLYR VIYELIDNVR
881 KAMEGLLETV EEQVPIGSAE VRAVFSSGSS RVAGCMVTDG KIVKDSGIRV LRKGKEVHVG VLSSLRRVKE LVKEVNAGLE
961 CGIGVEEFDD WEEGDRIEAF NTVQKKRTLE EASASMTAAL EGVGIEL

Supporting Peptides:

Peptide	Uniq	-10lgP	Mass	Length	ppm	m/z	z	RT	Fraction	Scan	Source File	Area Sample 1	#Feature	#Feature Sample 1	Start	End	PTM	AScore	Found By
V.VCGEAFGKA.R	Y	31.36	880.4113	9	-8.2	441.2060	2	33.38	1	6569	P2860-Aislado informe 2.raw	4.1833E6	1	1	691	699			PEAKS DB
**total 1 peptides**																			

Best Unique PSM (Scan 6569, m/z=441.2060, z=2, RT=33.38, ppm=-8.2):

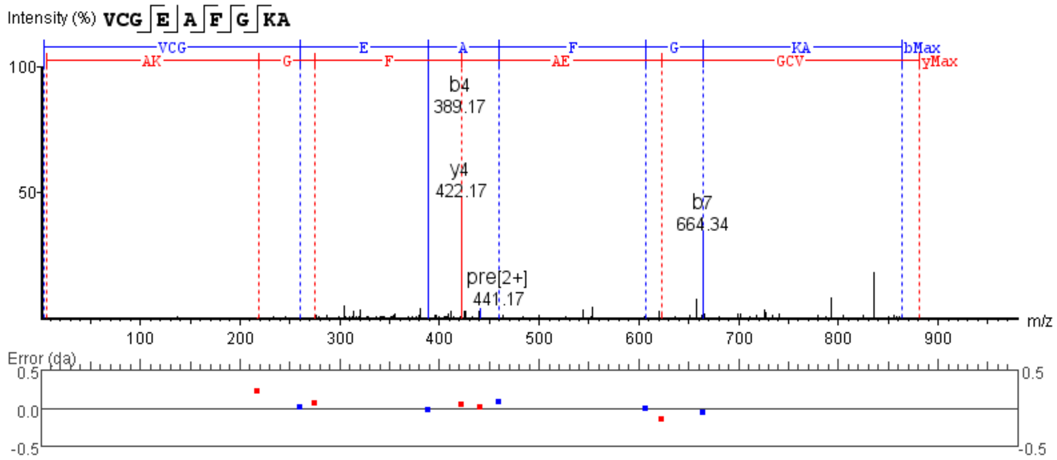

OE9A054657P1

back to list

| Protein Coverage | Supporting Peptides | Best Unique PSM |

Protein Coverage:

1 MPLLKEEPAD LEGSLAVTPS PPPQTGALVA APSKRSSRDR HTKVEGRGRR IRMPAACAAR IFQLTRELGH KSNDETIRWL
81 LERAEPAIIE ATGTVPPSPS PSTRQKRAQS SEFYDVTNSS NLAPVAPIAP QGLVPVFPSG TFFMIPPTAG AATIAAAGLS
161 NQPQFWAIPA TATPVFSISG RPISNFVSAM QAGVNMSFST STTTTTTTIN PTAVGGGAAA SSGNTESGEN SGGKISISTT
241 MAPSSSSVTS KTTVATGTQT LRDFSLEIYD KKELQIMSG**S GPGNHEQ**TPS SKP

Supporting Peptides:

Peptide	Uniq	-10lgP	Mass	Length	ppm	m/z	z	RT	Fraction	Scan	Source File	Area Sample 1	#Feature	#Feature Sample 1	Start	End	PTM	AScore	Found By
G.SGPGNHEQ.T	Y	30.94	824.3413	8	-6.5	413.1722	2	30.38	1	5739	P2860-Aislado informe 2.raw	0	0	0	280	287			PEAKS DB
**total 1 peptides**																			

Best Unique PSM (Scan 5739, m/z=413.1722, z=2, RT=30.38, ppm=-6.5):

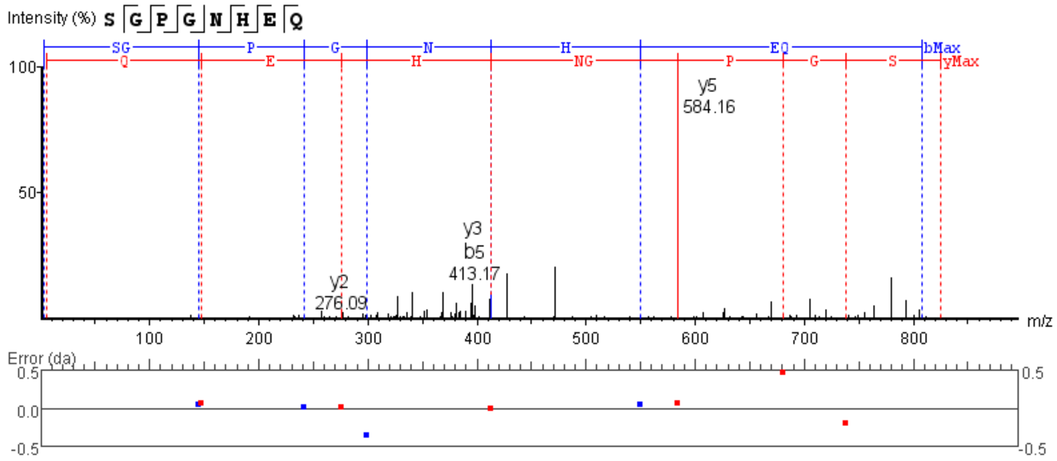

OE9A104108P1

back to list

| Protein Coverage | Supporting Peptides | Best Unique PSM |

Protein Coverage:

```
  1  MASIHRNHHS QPQHHHHHHS PPHIERQEHD EVITLPSDPL LGESHEYTIT AFGPPPDAAE TNPSPPPLPP PSSDIMPLLK
 81  EEPADLEGSL AVTPSPPPQT GALVAAPPKR SSRDRHTKVE GRGRRIRMPA ACAARIFQLT RELGHKSDGE TIRWLLERAE
161  PAIIEATGTG TVPAIAVSVN GTLKIPTTPS TASTSPETAG DGARKRRKRA QSSEFYDVAN SSNLAPVAPI APQGLVPLFP
241  SGTFFMIPPT AGAATIAAAG PSNQPQFWAI PATATPVFSI SGRPMSNFVS AMQAGVNMSF STSSTTTTIN PTAVGGGAAA
321  SSGNTESGEN SGGKISISTT MAPSSSSVTS KTTVATGTQT LRDFSLEIYD KKELQFMSGS GPGNHEQTPS SKP
```

Supporting Peptides:

Peptide	Uniq	-10lgP	Mass	Length	ppm	m/z	z	RT	Fraction	Scan	Source File	Area Sample 1	#Feature	#Feature Sample 1	Start	End	PTM	AScore	Found By
G.SGPGNHEQ.T	Y	30.94	824.3413	8	-6.5	413.1722	2	30.38	1	5739	P2860-Aislado informe 2.raw	0	0	0	380	387			PEAKS DB
**total 1 peptides**																			

Best Unique PSM (Scan 5739, m/z=413.1722, z=2, RT=30.38, ppm=-6.5):

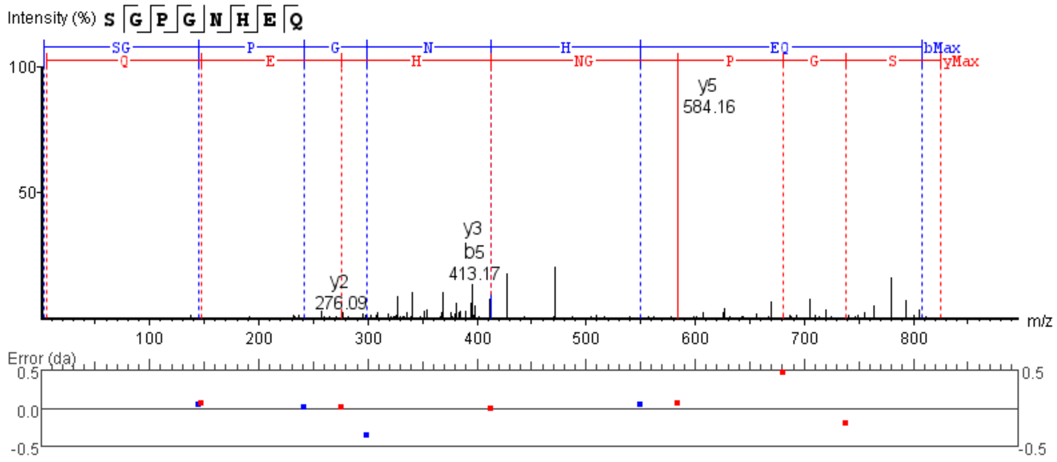

OE9A026632P1

back to list

| Protein Coverage | Supporting Peptides | Best Unique PSM |

Protein Coverage:

```
  1  MKNTSSGDAE GSSPNSTNPP QQLDQSSPTS SPLRRPPMAP SVMGNLGHSG FRPYRPQGRQ QFDQSSPQRG SNSNISSPMQ
 81  RPPVAPPPPP PPPLPLPVIG HTGQSAFMAH WPQGPQPQRQ FLGGGGSSGG AACASGNASS NGLFGAQAIP VGRSGGYRFG
161  GVGIGNPVIR KRVKEGNQGG QAEGGNPFQC RVCGKQFQSA KALFGHMRQL PDRGWKGTFP PPSFRAEEEF AEVPGHLNPG
241  RAAAAGDAAA AQAGEEATEV HEGREEAGDG LVGPQNYKLP DLNYKPPHDD HDAAA
```

Supporting Peptides:

Peptide	Uniq	-10lgP	Mass	Length	ppm	m/z	z	RT	Fraction	Scan	Source File	Area Sample 1	#Feature	#Feature Sample 1	Start	End	PTM	AScore	Found By
F.LGGGGSSGGAAC.A	Y	30.30	892.3708	12	-3.4	447.1878	2	20.67	1	2916	P2860-Aislado informe 2.raw	4.3063E5	1	1	122	133			PEAKS DB
**total 1 peptides**																			

Best Unique PSM (Scan 2916, m/z=447.1878, z=2, RT=20.67, ppm=-3.4):

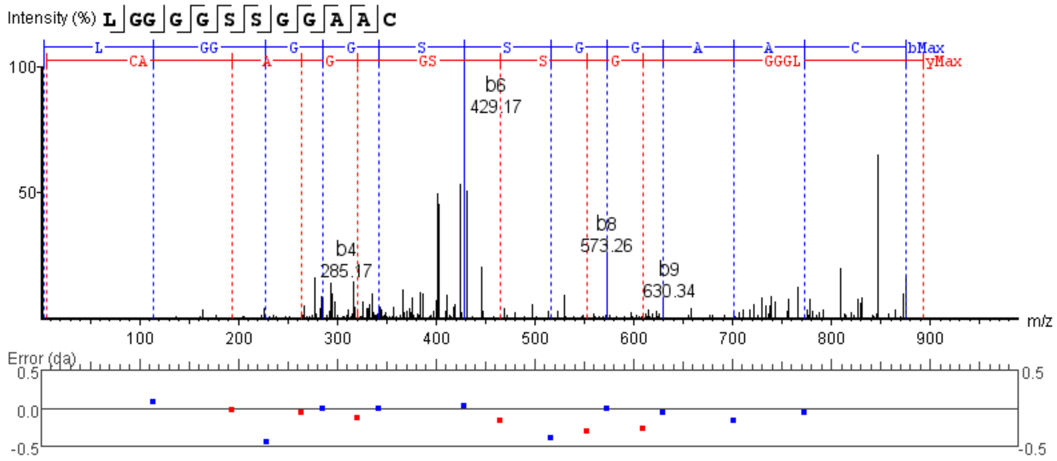

OE9A100476P1

[back to list]

| Protein Coverage | Supporting Peptides | Best Unique PSM |

Protein Coverage:

```
  1  MVTAKESCQQ LSAWNLPNLD TLLQLRHNNS LSCFPNSSSY AANVGFPGHS ICNLPGLKTG QLNRSDGFFQ NLPPCWESSS
 81  PIINPYLKDS QFGLSHGIGM SMNPAGSSST SQKKFLIFDQ SGNNTRLFFS PSFFPSQNQI IASTAAAGDH GTLDEVAVQT
161  EQEQQSLVKP VVQEKWDENH LTVGSEVRED TEEINALLCS NSTDEYDEVT SMGHTPFTIE ERYDKDIQIG ELVEEVASSN
241  GSTKRQKLLD GGYKKSSLVD MGDPLEMASP QNYEDGEESS CAGENNFYDD IDSIKKVKKV KIRESLKILE SIIPGLKNKD
321  PLLIIDRAII YLNSLKLEAE TLGISYL
```

Supporting Peptides:

Peptide	Uniq	-10lgP	Mass	Length	ppm	m/z	z	RT	Fraction	Scan	Source File	Area Sample 1	#Feature	#Feature Sample 1	Start	End	PTM	AScore	Found By
I.NALLCSNS.T	Y	30.09	820.3749	8	8.1	411.1950	2	27.63	1	4927	P2860-Aislado informe 2.raw	2.2457E5	1	1	195	202			PEAKS DB
total 1 peptides																			

Best Unique PSM (Scan 4927, m/z=411.1950, z=2, RT=27.63, ppm=8.1):

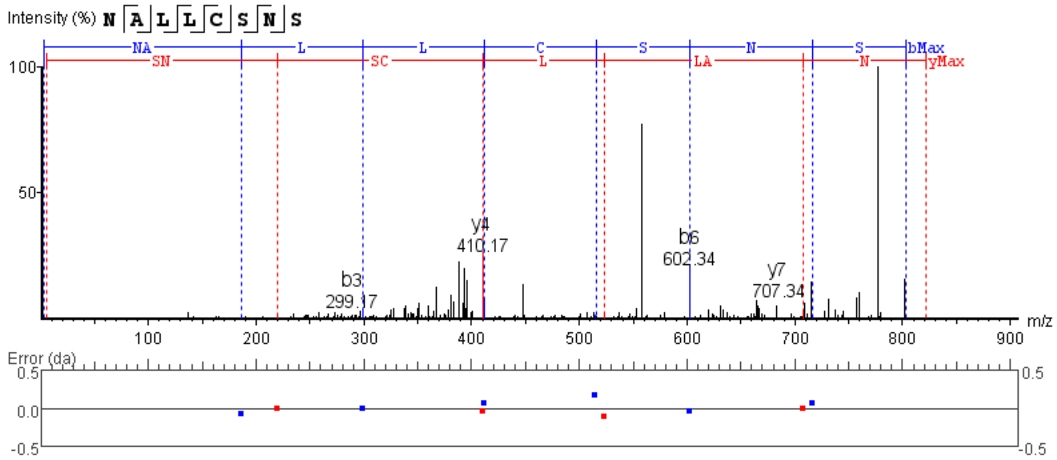

OE9A087887P1

[back to list]

| Protein Coverage | Supporting Peptides | Best Unique PSM |

Protein Coverage:

```
  1  MKTFVLVLLC LSSLMGALAQ GGVGSIISKS IFDQMLKHRN DANCPANGFY TYEAFIAAAN SFGAFGTTGD TDTRKREVAA
 81  FLAQTSHETT GGWASAPDGP YAWGYCHKQE EGNPPDYCVA NQQWPCAPGK KYFGRGPIQI SYNYNYGPAG RAIGSDLLNN
161  PDAVANDPTI SFKTALWFWM TPQTPKPSCH DAISGRWRPS AADSTAGRLP GYGVVTNIIN GGIECGKGSN PQVEDRIGFY
241  KRYCDLLGVG YGPNLDCYNQ RPFA
```

Supporting Peptides:

Peptide	Uniq	-10lgP	Mass	Length	ppm	m/z	z	RT	Fraction	Scan	Source File	Area Sample 1	#Feature	#Feature Sample 1	Start	End	PTM	AScore	Found By
N.CPANGFY.T	Y	29.54	770.3057	7	6.7	386.1598	2	26.19	1	4510	P2860-Aislado informe 2.raw	0	0	0	44	50			PEAKS DB
total 1 peptides																			

Best Unique PSM (Scan 4510, m/z=386.1598, z=2, RT=26.19, ppm=6.7):

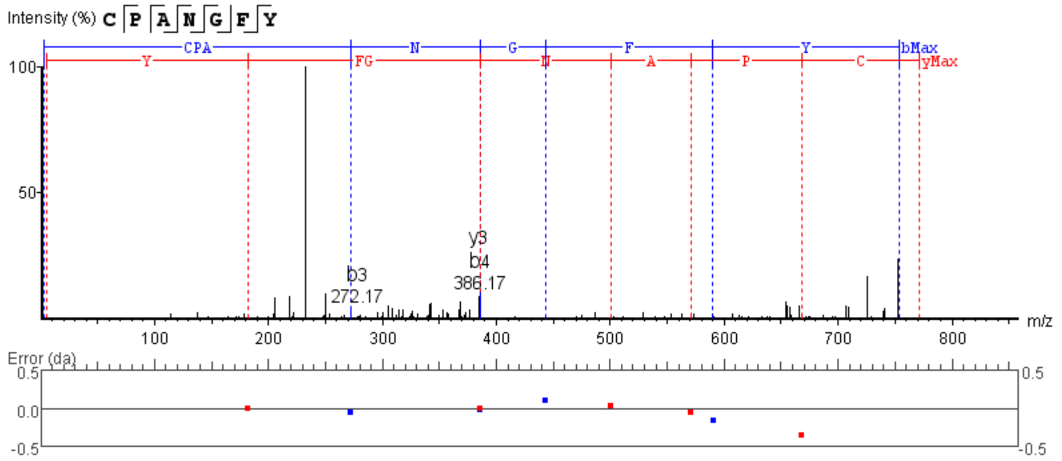

Peptide List

Prepared with PEAKS ™ (bioinfor.com)
