# Supplementary material for: Characterisation of Endogenous Peptides Present in Virgin Olive Oil
Source: Int J Mol Sci. 2022 Feb 2;23(3):1712. doi: 10.3390/ijms23031712 (PMC8836281; doi:10.3390/ijms23031712)
Supplement: Supplementary file 1 [file ijms-23-01712-s001.zip › 1. Supplemental material Table S1.pdf]

Supplemental material Table S1. Endogenous peptides identified in VOO olive oil using Peaks database (DB) algorithm, filtered by -10lgP score >25 (32 peptides). -10lgP score, statistical significance of the peptide-precursor spectrum match; N, number of amino acids; m/z, precursor mass-to-charge ratio; Mass, peptide mass in Da; RT, retention time; ppm, precursor mass error; Area, peptide abundance shown as area under the curve of the detected peptide; Accession, the accession number of the highest-scoring protein containing this peptide. Oxidised methionine is indicated by a pair of parentheses enclosing the modification mass (+15.99).

No.	Sequence	-10lgP	n	m/z	Mass	RT	ppm	Area	Accession
1	LDTANEMNQLDLQFR	93.56	15	904.4365	1806.8571	34.18	8.3	1.08E+06	OE9A085162P3:OE9A085162P1:<br>OE9A085162P2
2	VVLQDTSNNVNQLD	77.73	14	779.8878	1557.7634	24.96	5.9	3.44E+05	OE9A032471P1:OE9A042009P1
3	VVLQDTSNNVNQLDDIPRRFFLA	63.26	23	892.1344	2673.3875	48.98	5.2	0	OE9A032471P1:OE9A042009P1
4	AVVPIWLQPDTPAR	57.79	14	781.9397	1561.8616	38.09	9.6	4.39E+05	OE9A001718P2:OE9A001718P1
5	IFSGGESSGQPR	57.53	12	611.2961	1220.5785	22.47	6.8	1.24E+05	OE9A032471P1:OE9A042009P1
6	DTSNNVNQLDDIPRR	46.89	15	586.2892	1755.8500	26.25	5.1	1.54E+05	OE9A032471P1:OE9A042009P1
7	NCSTSIISG	39.01	9	441.2062	880.3961	35.88	9.5	3.49E+07	OE9A087501P1
8	VHVFRFDQNQDLLPIGN	34.71	17	671.3506	2011.0275	48.03	8.8	1.53E+04	OE9A001718P2:OE9A001718P1
9	LLLGAGCM(+15.99)	34.51	8	397.1985	792.3874	33.76	1.4	1.24E+07	OE9A117792P1
10	QDTSNNVNQLDDIPRR	33.36	16	628.9771	1883.9086	26.36	8.0	2.06E+06	OE9A032471P1:OE9A042009P1
11	FSASTEGS	32.74	8	393.1665	784.3239	25.27	0.5	9.63E+05	OE9A013295P1:OE9A042038P1:<br>OE9A062197P1
12	INTISGR	31.90	7	380.7197	759.4239	20.53	8.6	1.38E+05	OE9A032471P1:OE9A042009P1
13	LDGNSSAR	31.66	8	410.1964	818.3882	18.63	-4.9	1.41E+05	OE9A106456P1
14	VCGEAFGKA	31.36	9	441.2060	880.4113	33.38	-8.2	4.18E+06	OE9A058866P1
15	KGGGGGSGSAGGGGS	31.14	15	525.2275	1048.4534	35.95	-4.9	4.01E+06	OE9A057915P1
16	SGPGNHEQ	30.94	8	413.1722	824.3413	30.38	-6.5	0	OE9A054657P1:OE9A104108P1
17	LGGGGSSGGAAC	30.30	12	447.1878	892.3708	20.67	-3.4	4.31E+05	OE9A026632P1
18	NALLCSNS	30.09	8	411.1950	820.3749	27.63	8.1	2.25E+05	OE9A100476P1
19	CPANGFY	29.54	7	386.1598	770.3057	26.19	6.7	0	OE9A087887P1
20	LKSHKCES	29.07	8	466.2317	930.4593	35.48	-3.8	1.87E+05	OE9A081468P1
21	LSQLECS	28.48	7	390.1821	778.3531	21.25	3.0	2.46E+05	OE9A013196P1:OE9A013196P3
22	HSLGGGTGSGMG	27.92	12	509.2202	1016.4346	31.22	-1.2	4.96E+04	OE9A118395P1:OE9A084266P1
23	SIPGDICDSGSL	27.69	12	582.2623	1162.5176	34.63	1.0	4.80E+05	OE9A102443P1
24	VVGAACLY	27.50	8	398.2053	794.3997	28.98	3.0	2.49E+05	OE9A003377P2
25	LPGPGNMGC	27.50	9	423.1788	844.3571	27.03	-9.3	1.57E+05	OE9A039431P1:OE9A039431P2:<br>OE9A039431P3
26	ILGICGAM(+15.99)	26.58	8	397.1978	792.3874	30.93	-0.6	3.28E+06	OE9A070714P1
27	IPWGHPS	26.31	7	397.1978	792.3918	35.62	-6.2	1.62E+05	OE9A056660P1:OE9A056660P2:<br>OE9A056660P3:OE9A056660P5
28	LLQGCYP	26.09	7	3971.985	792.3840	25.33	5.6	0	OE9A103223P2:OE9A103223P1:<br>OE9A043277P1

29	YSLLCGA	25.78	7	363.6724	725.3418	31.05	-8.5	1.10E+05	OE9A046079P2
30	GGSGGGGAGAGAGAGG	25.64	16	509.2130	1016.4271	26.53	-8.0	1.12E+06	OE9A050974P2
31	NHHLSGL	25.53	7	389.1993	776.3929	34.78	-4.0	4.96E+04	OE9pt000020P1
32	DGAITGGSGGGS	25.17	12	468.2006	934.3992	19.26	-6.0	1.57E+04	OE9A076082P4:OE9A076082P5: OE9A076082P1
