# Supplementary material for: Characterisation of Endogenous Peptides Present in Virgin Olive Oil
Source: Int J Mol Sci. 2022 Feb 2;23(3):1712. doi: 10.3390/ijms23031712 (PMC8836281; doi:10.3390/ijms23031712)
Supplement: Supplementary file 1 [file ijms-23-01712-s001.zip › 1. Supplemental material Table S3.pdf]

Supplemental material Table S3. Endogenous peptides identified in VOO olive oil by de novo sequencing with ALC value above 80% (63 peptides). ALC, average local confidence; N, number of amino acids; m/z, precursor mass-to-charge ratio; Mass, peptide mass in Da; RT, retention time; Area, peptide abundance shown as area under the curve of the detected peptide; ppm, precursor mass error; local confidence, local confidence score for each amino acid of the peptide identified by de novo sequencing. Oxidised methionine is indicated by a pair of parentheses enclosing the modification mass (+15.99).

No.	Peptide	ALC (%)	N	m/z	Mass	RT	Area	ppm	local confidence (%)
1	CCYSVY	95	6	369.1365	736.256	24.36	5.77E+05	3.2	92 97 99 93 92 98
2	DCHYFL	94	6	399.1715	796.3214	25.13	6.41E+04	8.9	92 98 98 98 90 93
3	LYPFAH	94	6	374.1952	746.3751	19.07	3.59E+05	1	99 99 99 85 89 93
4	SVSKPGW	92	7	380.7006	759.3915	14.03	1.85E+05	-6.5	93 97 99 99 89 78 93
5	LHTVVH	91	6	353.2084	704.397	26.06	8.67E+04	7.6	99 93 93 92 85 87
6	NKLCCEH	91	7	423.6815	845.3524	29.15	0	-4.7	84 95 98 94 86 94 89
7	DHHEEL	91	6	390.1661	778.3246	36.35	7.71E+04	-8.9	89 91 97 91 92 85
8	LPCAAHR	90	7	384.2062	766.3908	19.09	2.70E+05	9.2	98 79 79 89 97 96 95
9	LALGAM(+15.99)DF	89	8	427.2069	852.4051	25.74	1.92E+06	-6.8	93 92 89 69 96 96 92 89
10	LMEAQR	89	6	374.1952	746.3745	19.07	3.59E+05	1.8	99 98 98 81 73 87
11	YDSDLR	89	6	384.6771	767.345	21.89	1.70E+05	-7	99 99 97 76 88 76
12	EM(+15.99)CPM(+15.99)Y	88	6	403.1284	804.2493	22.84	0	-8.7	90 78 87 82 95 98
13	HCLPSSH	88	7	390.6767	779.3384	44.29	0	0.5	93 96 99 77 84 80 85
14	LYNDVR	87	6	390.2079	778.3973	20.79	8.11E+05	5.1	99 99 93 85 76 75
15	MFLSYNY	87	7	469.2099	936.4052	19.37	0	0.2	85 87 96 86 88 79 87
16	DYSHMESH	86	8	503.1901	1004.3658	28.15	1.91E+05	-0.1	74 71 84 82 93 98 93 90
17	EMHHDQ	85	6	398.6558	795.297	24.99	3.81E+05	0	98 97 83 88 71 76
18	YQAHHA	85	6	363.6714	725.3245	28.15	4.20E+05	5.1	88 82 89 95 70 88
19	LKSECCY	85	7	423.1809	844.3459	29.08	0	1.7	95 93 95 96 76 69 72
20	YAHENTP	85	7	416.189	830.3559	16.36	7.15E+05	9.1	93 94 92 89 75 78 73
21	NSSYAGFR	85	8	451.2076	900.4089	18.19	7.11E+03	-9.2	82 92 88 79 88 71 95 86
22	DFMPDY	84	6	394.1516	786.2894	18.83	0	-0.9	85 86 94 76 81 86
23	DTLSAGMH	84	8	416.189	830.3593	16.36	7.15E+05	5	88 93 97 96 91 56 84 71
24	NSCAMRA	84	7	376.6606	751.3105	19.64	0	-5.3	73 82 82 88 98 82 87
25	HQSDNAECKM	84	10	581.7358	1161.4543	41.36	0	2.4	85 90 95 78 69 92 92 88 81 75
26	YCMMSERH	84	8	528.707	1055.3987	32.72	0	0.8	95 98 97 94 73 80 67 72
27	HM(+15.99)VHQT	84	6	384.6764	767.3385	25.3	1.10E+05	-0.4	74 86 93 93 71 88
28	LYLAMH	84	6	374.1952	746.3785	19.07	3.59E+05	-3.5	98 99 90 78 66 74
29	LPACEGCL	84	8	403.1799	804.351	26.08	0	-7.2	91 78 77 85 95 62 92 93
30	YEHSWH	84	6	429.6818	857.3456	41.36	0	3.9	75 91 77 84 80 98

Supplemental data 2 (cont).

No.	Peptide	ALC (%)	N	m/z	Mass	RT	Area	ppm	local confidence (%)
31	FVESQGS	84	7	377.1768	752.334	25.85	4.75E+05	6.5	97 95 98 85 80 57 75
32	LYLFSH	83	6	390.2083	778.4014	21.28	4.35E+05	0.9	88 93 86 73 82 79
33	VLSSSGRYGGP	83	11	540.279	1078.5408	22.84	7.27E+04	2.5	95 98 98 95 75 45 66 85 95 82 82
34	NCCAPKF	83	7	391.6669	781.3251	29.62	0	-7.6	68 79 99 99 74 70 90
35	KAHSAYD	83	7	396.1902	790.361	20.79	1.18E+05	6.1	76 77 82 98 91 79 76
36	FLGSAEW	83	7	405.1957	808.3755	21.15	2.46E+05	1.6	70 80 79 90 77 89 94
37	LLEM(+15.99)HANY	83	8	503.7346	1005.459	38.13	0	-4.2	92 87 98 95 87 52 79 70
38	YDCGLFN	83	7	416.1675	830.3268	21.5	0	-7.7	89 85 85 62 91 83 83
39	YCFCMY	83	6	415.1432	828.2645	31.37	0	8.9	68 74 92 92 83 87
40	LCGPLCH	82	7	371.6688	741.3302	27.24	1.38E+06	-9.7	96 90 52 60 95 89 93
41	KAHCLM(+15.99)Y	82	7	441.2062	880.3936	35.88	3.49E+07	4.9	66 75 95 99 89 73 79
42	DM(+15.99)RRVM(+15.99)	82	6	420.2005	838.379	23.83	3.15E+05	8.9	69 78 85 88 78 95
43	HCGSLDH	82	7	384.6577	767.3021	28.11	1.20E+05	-1.5	85 85 83 88 82 73 78
44	LRDDPR	82	6	386.2092	770.4034	20.54	0	0.4	92 71 74 79 79 95
45	HCGHDCAE	81	8	436.1471	870.2748	22.91	2.75E+05	5.6	57 68 78 90 96 89 86 90
46	LELHAGEY	81	8	466.2323	930.4446	29.38	5.75E+05	5.7	88 97 97 79 90 45 87 69
47	YDVSMVSE	81	8	465.1997	928.3848	32.16	0	0	89 86 89 97 85 61 63 79
48	VVSCSCHCPY	81	10	549.2114	1096.4141	32.91	5.35E+06	-5.3	78 80 86 93 84 88 86 88 61 66
49	YCARCCCE	81	8	475.6541	949.2914	19.59	1.39E+04	2.4	85 77 85 82 95 82 70 70
50	LYPAQME	81	7	426.2006	850.3895	26.25	8.21E+05	-3.4	93 89 85 87 85 54 73
51	LKSHCQES	81	8	466.2209	930.4229	35.65	0	4.8	93 91 92 88 60 62 92 69
52	DCHPPRS	81	7	406.1794	810.3442	26.18	1.49E+05	0	92 96 98 91 66 51 71
53	LQAAYDS	80	7	384.1851	766.3497	28.92	9.15E+05	7.8	76 60 61 86 98 93 91
54	YLQPCSP	80	7	404.1874	806.3633	17.96	1.49E+06	-3.7	84 86 86 72 87 76 73
55	LNAPVMM(+15.99)	80	7	396.1902	790.3718	20.79	1.18E+05	-7.5	79 65 85 78 92 82 82
56	AM(+15.99)PPGGVAH	80	9	426.7036	851.3959	31.32	0	-3.8	88 90 92 89 60 59 67 90 86
57	LLYAHY	80	6	390.2085	778.4014	28.41	6.58E+05	1.3	99 99 95 63 57 67
58	YAATCSY	80	7	389.6585	777.3004	21.25	2.41E+05	2.7	87 95 90 86 57 59 88
59	FNSCVHAQ	80	8	453.1988	904.3861	26.63	8.20E+04	-3.5	79 79 88 86 92 68 76 73
60	DHDAYD	80	6	368.1342	734.2507	22.54	0	4.3	66 75 87 85 82 83
61	NCYHCCAKVP	80	10	569.2372	1136.4565	31.89	6.67E+05	2.9	70 90 96 94 95 90 74 68 71 51
62	LKHSGLCAC	80	9	466.2317	930.4415	35.48	1.87E+05	7.8	93 94 97 97 82 68 50 70 67
